# Interplay of ecological processes modulates microbial community reassembly following coalescence

**DOI:** 10.1093/ismejo/wraf041

**Published:** 2025-04-03

**Authors:** Luana Bresciani, Gordon F Custer, David Koslicki, Francisco Dini-Andreote

**Affiliations:** Department of Plant Science, The Pennsylvania State University, University Park, PA 16802, United States; Huck Institutes of the Life Sciences, The Pennsylvania State University, University Park, PA 16802, United States; One Health Microbiome Center, The Pennsylvania State University, University Park, PA 16802, United States; Department of Plant Science, The Pennsylvania State University, University Park, PA 16802, United States; Huck Institutes of the Life Sciences, The Pennsylvania State University, University Park, PA 16802, United States; One Health Microbiome Center, The Pennsylvania State University, University Park, PA 16802, United States; Department of Natural Sciences, The University of Maryland Eastern Shore, Princess Anne, MD 21853, United States; Huck Institutes of the Life Sciences, The Pennsylvania State University, University Park, PA 16802, United States; One Health Microbiome Center, The Pennsylvania State University, University Park, PA 16802, United States; Department of Computer Science and Engineering, The Pennsylvania State University, University Park, PA 16802, United States; Department of Biology, The Pennsylvania State University, University Park, PA 16802, United States; Department of Plant Science, The Pennsylvania State University, University Park, PA 16802, United States; Huck Institutes of the Life Sciences, The Pennsylvania State University, University Park, PA 16802, United States; One Health Microbiome Center, The Pennsylvania State University, University Park, PA 16802, United States

**Keywords:** invasion biology, soil microbiology, species interactions, abiotic filtering, community assembly, beta-diversity

## Abstract

Microbial community coalescence refers to the mixing of entire microbial communities and their environments. Despite conceptually analogous to a multispecies invasion, the ecological processes driving this phenomenon remain poorly understood. Here, we developed and implemented a beta-diversity–based statistical framework to quantify the contribution of distinct donor communities to community reassembly dynamics over time following coalescence. We conducted a microcosm experiment with soils manipulated at varying levels of community structure (via dilution-to-extinction) and subjected these to pairwise coalescence scenarios. Overall, our results revealed variable patterns of abiotic and biotic donor dominance across distinct treatment sets. First, we show the occasional presence of an upfront stringent abiotic filter to disproportionally favor a donor biotic dominance through a “home-field advantage” mechanism, with abiotic factors explaining >90% of the variance in community structure. Functional community metrics (i.e. carbon metabolism and extracellular enzymatic activities) were significantly linked to donor contributions in these cases. Second, in the absence of abiotic dominance, interspecific interactions gained importance, with abiotic variables explaining <40% of the variance. Here, functional redundancy in donor communities (e.g. lower dilution) led to nonsignificant relationships between donor contributions and functional metrics. Collectively, this study advances the integration of coalescence with well-established fundamentals of invasion biology theory, highlighting the interplay of abiotic and biotic factors structuring community reassembly following coalescence. Last, we propose that our beta-diversity–based framework is widely applicable across various microbial systems. We believe this approach will promote research advances by offering a unified method for analyzing and quantifying coalescence.

## Introduction

Microbial communities frequently disperse and invade other communities as intact ecological units—an ecological phenomenon known as “community coalescence” (*sensu* [1]). This process of microbial community coalescence (MCC) can be observed in natural ecosystems (e.g. flooding of coastal areas, river confluences, animal fecal droppings), during social human interactions (e.g. handshaking, intimate kissing), in agricultural system management practices (e.g. bioorganic amendments), and in medical treatments (e.g. fecal microbiota transplantation) [2–6]. Over the past decade, the study of MCC has garnered attention, with research efforts focused on elucidating the complex interplay of ecological mechanisms modulating species engraftment following MCC. Collectively, these studies mostly aimed to provide a better understanding of the direct consequences of MCC for microbial-mediated (eco)system functioning and performance [6, 7].

A comprehensive overview of the current literature on MCC shows that the majority of the studies have focused on the following topics: (i) exploring the dynamics of species turnover and engraftment over time [8–10], (ii) quantifying patterns of species interactions (e.g. competition and cooperation) upon community mixing [11, 12], (iii) describing aspects of species coselection and ecological cohesiveness [13], (iv) determining the influence of community diversity on resource utilization affecting community reassembly [14, 15], and (v) developing strategies to model the functional outcomes of species engraftment following MCC [16]. Despite these advances, we still lack a comprehensive understanding of how distinct ecological processes interact to determine the temporal dynamics of community reassembly following MCC.

In a recent study, we developed a conceptual synthesis that integrates MCC with principles of invasion biology theory [6]. In brief, we reasoned patterns of community reassembly to be determined by the interaction of community biotic and abiotic mixing, leading to temporal dynamics in community reassembly. These dynamics can also alter the relative contribution of each donor community to the outcome community over time (i.e. donor dominance). Of key importance, we also advocated the need for the development of quantitative frameworks to study the shifts in the relative influence of biotic and abiotic variables on community reassembly following MCC. In addition, we further conceptualized the level of biological diversity and abiotic properties in a system to directly influence the outcome of species engraftment, mostly due to variation in resource availability and resource use efficiency across distinct species following MCC (in line with the “diversity-invasion relationship,” see [17, 18]).

Here, we developed and implemented a framework able to quantify the relative contribution of individual microbial communities (i.e. donors) to the outcome community following MCC. This framework parametrizes community structure (based on species taxonomic information) and functional profiles (e.g. carbon metabolism and enzymatic activities) and can be implemented using time-series analysis to track shifts in the donors’ relative contribution to community reassembly following MCC. We then performed a microcosm experiment in which distinct ecological communities were manipulated at different levels of species composition (i.e. using a dilution to extinction approach—see below) and subjected to MCC. We quantified the relative contribution of donors to the outcome community over time and sought to answer the following questions: (i) Does the relative contribution of donor communities vary over time following MCC (at both structural and functional levels)? (ii) Does disruption in community structure affect the relative dominance of donor communities determining community reassembly? and (iii) To what extent, does abiotic dominance determine the outcome of community reassembly following MCC? In line with these questions, we hypothesized (i) mixing of distinct communities in equal proportions to result in dynamic patterns of donor dominance over time mediated by an interplay of biotic and abiotic filters following coalescence; (ii) variation in species composition and community structure (i.e. biotic dilutions causing stochastic disruptions in species abundances and ecological interactions—see below) to exert an effect on donor contributions to the outcome communities; (iii) the existence of dominant abiotic factors in a donor and outcome community to favor biotic dominance due to “home-field advantage” (i.e. constant abiotic species selection) with direct impacts on functional community metrics. In this study, we applied this quantitative framework to time-series data assessing bacterial community structure and functional profiles collected from distinct MCC scenarios. The quantitative approach implemented in this study has the potential to be broadly applicable across various microbial ecology subdisciplines, including human microbiome and environmental microbiology research, and aid in the development of predictive models for species engraftment and functional outcomes following MCC.

## Materials and methods

### Soil collection and sterilization

Soils were collected from field sites located in DE (Soil A, coordinates: 38° 32′ 29.0508″ N, 75° 41′ 57.156″ W), MI (Soil B, coordinates: 42° 58′ 24.6756″ N, 85° 49′ 52.068″ W), and NE (Soil C, coordinates: 41° 16′ 36″ N, 96° 00′ 42″ W), USA, in the Spring of 2022. Soils were selected based on their relatively low clay content to facilitate mixing during experimental manipulations (3.03 ± 0.7, 6.97 ± 0.65, and 29.33 ± 0.7%, respectively) and the expected differences in physicochemical and biological properties (Supplementary Tables S1 and S2). Briefly, soil samples were collected from the top 10 cm after removing the litter layer using sterile tools, transported to the laboratory (<24 h), and stored at 4°C. A subset of these soil samples (i.e. 10 kg per sample) was sterilized via gamma-irradiation (>35 kGy) at the Penn State Gamma Irradiation Facility (Cobalt-60 Pool Irradiator). Soil sterilization was confirmed by streak plating on Tryptic Soy Agar plates. The remaining nonsterilized soils (herein referred to as “natural soils”) were kept at 4°C for nutrient analysis and used as inoculum sources subjected to serial dilutions and inoculated in sterile soils (see below for details). Soils were subjected to physicochemical analyses (i.e. determination of clay, sand, and silt contents, soil pH, potential acidity, phosphorus [P], potassium [K], calcium [Ca], magnesium [Mg], zinc [Zn], copper [Cu], sulfur [S], cation exchange capacity [CEC], organic matter [OM], total carbon [TC], soluble salts, nitrate [N-NO_3_^−^], ammonium [N-NH_4_^+^], and total nitrogen [TN]) (for details, see Supplementary Methods).

### Microcosm manipulations: Soil serial dilutions and inoculations

To manipulate the biotic structure of microbial communities in each soil, gamma-irradiated Soils A–C were reinoculated with serial dilutions from their respective microbial communities. For that, 10 g of each natural soil was serially diluted in sterile DI water (1:10) from 10^−1^ to 10^−5^. Sterile soils were inoculated with one of three different biotic dilution levels (10^−1^, 10^−3^, or 10^−5^), and each level was treated independently for the remainder of the experiment. A volume of 10 ml of each inoculum was added to 100 g of sterile soil (1:10) in three replicate bags. Three independent replicates for each of the three levels of serial dilution (10^−1^, 10^−3^, 10^−5^) per soil were performed to capture variability during soil recolonization. After inoculation, soil moisture was adjusted to 65% water holding capacity (WHC) with sterile DI water. These inoculated soils were incubated at 25°C for 60 days to promote soil recolonization. Bags were closed with sterile cheesecloth to allow for gas exchange. Bags were weighed every 7 days, and moisture loss was estimated and replaced with sterile DI water. Following the 60-day incubation, replicate bags of each dilution and soil type were homogenized, and the soil was reincubated for an additional 7 days to allow for a final community stabilization (for further details, see Supplementary Methods and Supplementary Fig. S2).

### Microcosm experiment and sample collection

Experimental manipulation of MCC was performed by mixing distinct soils at the same dilution level (e.g. Soil A 10^−1^ with Soil B 10^−1^ or Soil C 10^−5^ with Soil B 10^−5^) in all possible combinations (Supplementary Fig. S1). Experimental factors (i.e. biotic dilution, soil type, and time after MCC) were considered in the experiment in a split–split plot design (SSPD). SSPD is suited for experiments in which different factors require different levels of restrictions or are set up as individual experimental units. Microcosms were constructed using 12.5 g of each donor soil (i.e. 1:1 soil mixing), mixed thoroughly using a sterile spatula and a vortex at maximum speed for 30 s. Controls for each dilution level consisted of 25 g of each individual soil from a single donor at the respective biotic dilution factor. Microcosms were covered with sterile cotton to allow gas exchange and incubated at 25°C in the dark for a total of 30 days. Microcosms were weighed on the day of experimental mixing, and moisture was replaced with sterile DI water every 5 days to maintain WHC at 65%.

Destructive sampling was performed on Days 1, 5, 15, and 30 following MCC, and samples were subjected to (*1*) total soil DNA extraction and amplicon sequencing analysis of bacterial communities, (*2*) determination of extracellular enzymatic activities, and (*3*) quantification of community carbon metabolism profiles based on Biolog EcoPlates™ (Biolog Inc., CA, USA) (see below). In total, we report 432 independent samples used in downstream analysis [3 soil pairs × 3 biotic dilution levels × 4 sampling points × 6 replicates] + [3 soil controls × 3 biotic dilution levels × 4 sampling points × 6 replicates]. Hereafter, we use the soil name (A–C), MCC scenarios (AxB, AxC, and BxC), and biotic dilution (10^−1^, 10^−3^, and 10^−5^) to refer to the treatment sets. The notation [Soil/MCC scenario]*^x^* refers to all biotic dilutions within a treatment set.

### Total soil DNA extraction and sequencing

Total soil DNA was extracted using the DNeasy PowerSoil Pro kit (QIAGEN, MD, USA), following the manufacturer’s instructions. DNA quality and concentration were determined using a Nanodrop One Microvolume UV–Vis spectrophotometer at A260/A280 nm (Thermo Fisher Scientific, MA, USA). DNA extracts were amplified using the modified primer 515F (5′-GTG YCA GCM GCC GCG GTA A-3′) [19] and 806R (5′-GGA CTA CNV GGG TWT CTA AT-3′) [20], targeting the V4 region of the bacterial 16S rRNA gene. The polymerase chain reaction (PCR) mix consisted of 0.25 μl of Phusion high-fidelity DNA polymerase, 5 μl of 5× Phusion Green HF buffer, 0.5 μl of deoxynucleoside triphosphates (10 mM), 17.75 μl of diethyl pyrocarbonate–water, 0.25 μl of each forward and reverse primers (10 μm), and 1 μl (ca. 10 ng) of template DNA. Thermocycler conditions were set at 94°C for 3 min, followed by 30 cycles at 94°C for 45 s, 50°C for 60 s, and 72°C for 90 s, with a final extension at 72°C for 10 min. PCR products were verified on a 1.5% agarose gel electrophoresis stained with SYBR Safe DNA gel stain (Invitrogen, CA, USA). PCR products were subjected to library preparation and sequencing at the Pennsylvania State University Genomics Core Facility. In brief, Illumina adapters and barcodes were added to PCR products using a two-step PCR. Amplicons were multiplexed and sequenced on an MiSeq System (Illumina) platform using the standard V2 reagent kit (2 × 250 paired-end sequencing).

### Amplicon sequencing analysis

Demultiplexed, raw sequence reads were processed in R (version 4.4.1) [21] using the DADA2 package v. 1.20 [22]. Processing consisted of quality filtering, error estimation, error correction, and merging of paired reads. Forward and reverse reads were truncated at 240 and 220 bp, respectively. Filter and trim parameters were set as follows: filterAndTrim (truncLen = c(240,220), maxEE = c(2, 2), truncQ = 2, maxN = 0, rm.phix = TRUE). Sequences were clustered into amplicon sequence variants (ASVs), followed by chimera removal using the removeBimeraDenovo method. Taxonomic assignments were performed against the Silva v.138.1 rRNA database [23, 24]. ASVs not assigned to ‘Bacteria’ and those assigned to mitochondria or chloroplast were removed. The final ASV table was rarefied using the EcolUtils R package v. 0.1 with the function rrarefy.perm() [25]. The final ASV table consisted of 15 000 reads per sample.

### Functional analyses

Extracellular enzymatic activities were determined for β-glucosidase, β-xylosidase, α-glucosidase, cellobiohydrolase, *N*-acetyl-β-glucosaminidase, leucine aminopeptidase, acid phosphatase, and sulfatase (see Supplementary Methods for details). In addition, carbon metabolism profiles were determined using Biolog EcoPlates™. In brief, Biolog EcoPlates™ contain 31 ecologically relevant and structurally variable carbon compounds in triplicate arranged in 96-well plates. For the assay, 1 g of soil was suspended in 9 ml of 0.85% NaCl solution and shaken at 25°C for 10 min at 150 rpm. Soil solutions were further subjected to 1000 × dilution. A volume of 125 μl was pipetted into each EcoPlate well and incubated at 25°C for 7 days in the dark. The consumption of individual carbon substrates was assessed via colorimetric change due to tetrazolium dye reduction from nicotinamide adenine dinucleotide production. Data were collected every 24 h (up to 168 h) by measuring the intensity of color change at an optical density (OD) of 590 nm in a BioTeck Synergy H1 Microplate reader. Four of the six replicates per treatment were randomly selected for Biolog assays for a total of 288 plates. The obtained OD measurements were normalized against the value of water-only control, followed by the subtraction of the first plate reading (Day 0) to eliminate the color effect of soil particles on OD values.

### Statistical analysis

Statistical analysis and visualizations were performed in R (version 4.4.1) [21] and Python 3.8 [26]. First, to test for differences in abiotic properties, one-way analysis of variance (ANOVA) followed by Tukey’s HSD was used. This was used to test for statistically significant differences in individual soil properties before and after gamma-irradiation and between the donors and mixtures (*α* = 0.05). Second, differences in abiotic properties between the donors and mixtures were visualized using principal coordinates analysis and tested using permutational multivariate analysis of variance (PERMANOVA) based on Euclidean distances. For that, pairwise distances were calculated using the “phyloseq” package in R [27]. Next, we used the Adonis function in the “vegan” package [28], with 999 permutations. This was done independently for each treatment set (i.e. A, B, and AxB; A, C, and AxC; and B, C, and BxC). Differences in abiotic properties among sets of donors and mixtures were also visualized using the *z*-score transformation of each abiotic variable and plotted using the heatmap.2 function in the “gplots” package in R [29]. Statistical differences in biotic properties before and after soil mixing were determined using ANOVA with Tukey’s HSD (e.g. Observed ASVs, Chao1, Shannon, and evenness indexes) (*α* = 0.05) (Supplementary Figure S3 and Supplementary Table S4). In addition, Venn diagrams of community mixtures were created using get_vennlist function on “MicrobiotaProcess” package in R [30] (Supplementary Figure S4).

### Development of a quantitative framework to study microbial community coalescence

**Figure 1:**
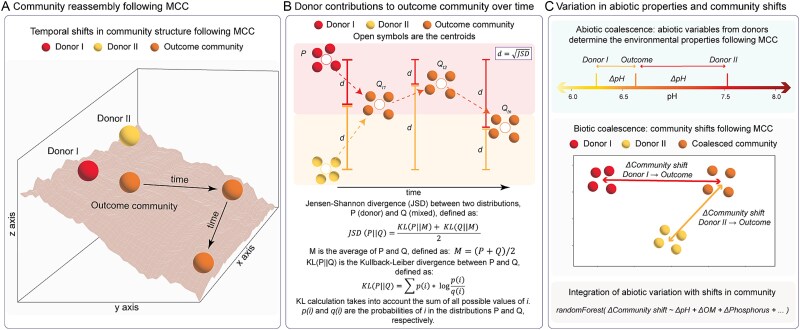
Overview of the framework used to quantify the relative contribution of donor communities to the outcome community following MCC. (A) Conceptual diagram depicting the temporal shifts in the outcome community following coalescence, (B) schematic view of the model used for quantifying the contribution of donor communities to the outcome community over time based on JSD distances, and (C) integration of variation in individual abiotic variables with the shifts in biotic community structure between the donors and outcome.

We developed a quantitative framework for the quantification of relative contributions of the donor microbial communities to the outcome community reassembly based on beta-diversity metrics (using both taxonomic and/or functional datasets). In brief, the framework uses a count table—a matrix where columns represent the samples and rows represent features (e.g. species, ASVs, operational taxonomic units (OTUs), or any other functional *n* * *m* count matrix)—to calculate pairwise distances between donors and mixtures using several independent distance metrics: including Jensen–Shannon divergence (JSD), Euclidean, Cosine, and Earth Mover’s distances. Pairwise distances are then organized into a matrix that allows for follow-up statistical testing (e.g. hierarchical clustering or ordination, providing a visual representation of the similarity between samples across different treatment groups and time points) (Fig. 1A). This framework integrates time-series data analysis to provide an assessment of the temporal shifts in community reassembly following MCC by analyzing the permuted pairwise distances between donors and outcomes at each time point. Briefly, the distances from Donor I to outcome and from Donor II to outcome are calculated within a sampling time point (Fig. 1B). All possible distances from donors to outcome within a sampling time point are calculated to characterize the distribution of donor-to-outcome distances (e.g. I_rep1_ to I * II_rep1…rep*N*_, I_rep2_ to I * II_rep1…rep*N*_). Here, smaller distances between the donor and the outcome indicate a greater donor contribution to the outcome community (Fig. 1B). To determine statistical significance in donor contributions and dominance, the distances are subjected to normality tests (e.g. Shapiro–Wilk). After that, comparative tests such as *t*-tests or Mann–Whitney *U* tests are performed, depending on the data distribution using *α* = 0.05 for statistical significance. Hereafter, “donor contribution” refers to the structural resemblance between each donor and the outcome community (e.g. community structure, functional structure), and “donor dominance” refers to the donor with the greater structural resemblance with the outcome community.

### Application of the quantitative framework on datasets obtained in the microcosm experiment

Linear regression analyses (“lm” function in base R) (JSD distance from donor to outcome community at *T_n_* ~ Time) were used to quantify the relative contribution (i.e. donor dominance) of donor communities to outcome community over time using the datasets of bacterial community structure, extracellular enzymatic activities, and carbon metabolism profiles. Further, correlational analyses were used to test whether differences in donor dominance in community structure were associated with donor dominance in carbon metabolism and enzymatic activities. This was performed by correlating the contribution of donor communities based on community structure data (i.e. donor dominance, as per JSD) with the pairwise JSD dissimilarity in functional profiles (e.g. enzymatic or metabolic) using Spearman correlation (JSD-determined community dominance ~ JSD-determined enzymatic/metabolic dominance).

To evaluate the importance of shifting individual abiotic properties in determining changes in bacterial community structure following MCC, we employed a regression approach (Fig. 1C). Here, we considered the absolute difference of each abiotic variable between donor and mixture (referred to here as “absolute delta”) (e.g. pH variation between Donor I and Outcome I * II, and between Donor II and Outcome I * II [Fig. 1C]) and the associated pairwise Bray–Curtis distances between each donor and outcome community independently for each biotic dilution and time point (Fig. 1C). The differences in abiotic properties and community dissimilarity following MCC were used to test for the association between variations in abiotic properties and community shifts. Here, we anticipate that small shifts in abiotic properties would coincide with small shifts in community composition and vice versa. To test this, we used random forest (RF) regression—a machine learning algorithm—to predict the importance of changes in sand, pH, OM, N-NH_4_^+^, N-NO_3_^−^, TN, and S for determining shifts in community structure (Fig. 1C). RF analysis was conducted using the “rfPermute” package in R [31], and each predictor was permuted for each tree, with an average of over 1000 trees. Factor importance was estimated using the increase of mean square error and *P*-values were calculated by permutating the response of community structure change (perm = 100).

## Results

### Physicochemical properties of donor soils and outcome soil environment

**Figure 2:**
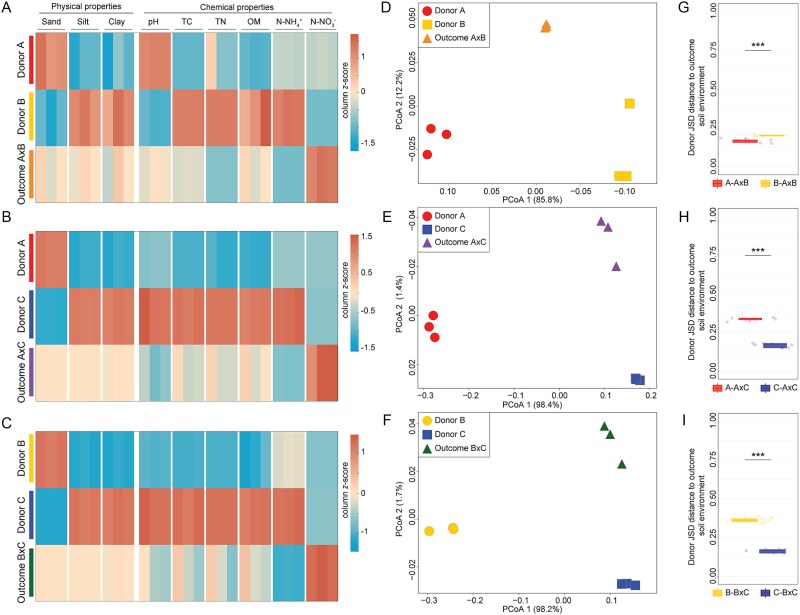
Physicochemical properties of donor and outcome soils. (A–C) Heatmaps displaying soil variables (e.g. sand, silt, clay, pH, TC, TN, OM, ammonium [N-NO_3_^−^], and nitrate [N-NH_4_^+^]) for treatment sets (A) A, B, and AxB; (B) A, C, and AxC; and (C) B, C, and BxC. Heatmaps are colored based on *z*-score transformations calculated individually for each abiotic factor. (D–F) Principal coordinate analysis plots based on Euclidean distances of abiotic properties for the treatment sets (D) a, B, and AxB; (E) a, C, and AxC; and (F) B, C, and BxC. (G–I) Contribution of donor soils to the outcome soil environment based on JSD distances for the treatment sets (G) AxB, (H) AxC, and (I) BxC. Statistical differences in the relative contribution of donors to outcome soils were determined using nonparametric Mann–Whitney *U* tests and are denoted by an asterisk above the boxplot (^***^P < .001). Lower distances represent a greater contribution of the donor to the outcome soil (i.e. dominance).

The physical and chemical properties of donor soils (i.e. A–C) were significantly different from each other at the start of the experiment (*P* < .05). Following the experimental manipulations of MCC—based on a 1:1 ratio—the physical properties (e.g. sand, silt, and clay %) of outcome soils (e.g. in AxB, AxC, and BxC) significantly differed from the donors (*P* < .05) (Supplementary Table S2). For soil chemistry, the donors differed significantly from each other (*P* < .05) in all chemical components (e.g. pH, potential acidity, P, K, Zn, Cu, S, CEC, OM, TC), except for Mg, Ca, soluble salts, and TN, since donors A and B had similar values but differed from Soil C (Supplementary Table S3). However, for donors and mixtures, chemical analysis revealed distinct patterns for soil parameters (e.g. average and deviations from the average values) (Supplementary Table S3). In brief, the outcome of AxB resulted in significant differences in soil pH, P, Cu, N-NO_3_^−^, and N-NH_4_^+^ and soluble salts contents when compared to donors A and B (*P* < .05), but the values of S, acidity index, CEC, and TC were statistically similar to those in Donor A, and concentration of Zn was statistically similar to the value in Donor B (*P* < .05) (Fig. 2A and Supplementary Table S3). Contents of OM, K, Mg, Ca, and TN were significantly similar to those in both donors (*P* > .05). Conversely, the outcome of AxC significantly differed from the donors for OM, P, K, Mg, Ca, Cu, Zn, S, N-NO_3_^−^, N-NH_4_^+^, soluble salts, CEC, TC, and TN, and soil pH (Fig. 2B and Supplementary Table S3). Last, the mixture of BxC displayed statistically different values of pH, OM, K, Mg, Ca, Zn, Cu, N-NO_3_^−^, N-NH_4_^+^, soluble salts, acidity index, CEC, TC, and TN as those observed in donors B and C (*P* < .05). Soil S content presented similar values to Donor B and soil P to Donor C (*P* > .05) (Fig. 2C and Supplementary Table S3).

PERMANOVA revealed the donors and outcome soil environments to be significantly different in soil physicochemical properties within treatment sets AxB (*F* = 81.5, *R^2^* = 0.96, *P* < .01), AxC (*F* = 305.9, *R^2^* = 0.99, *P* < .01), and BxC (*F* = 143.6, *R^2^* = 0.98, *P* < .01) (Fig. 2D–F). The relative contribution of the donor soils to the outcome soil environment revealed consistent results across multiple metrics (i.e. JSD, Cosine, Euclidean, and Earth Mover’s distances; Fig. 2G–I and Supplementary Fig. S5 and Supplementary Table S5). Only the results from the JSD distances are shown in the main text as similar patterns were observed across metrics (see Supplementary Figs. S5–S8 and Supplementary Tables S5–S8 for details). In brief, the results based on JSD distances for the treatment set AxB showed a greater contribution of Donor A (0.17 ± 0.01) over Donor B (0.20 ± 0.01) to the outcome soil environment (*P* < .001). However, for the treatment set AxC, Donor C had a greater contribution to the outcome soil environment (0.17 ± 0.01) when compared to Donor A (0.34 ± 0.01) (*P* < .001). This dominance of Donor C was also observed in the treatment BxC, with Donor C having a greater contribution to the abiotic environment (0.16 ± 0.01) than Donor B (0.36 ± 0.02) (*P* < .001) (Fig. 2G–I, see Supplementary Information and Supplementary Table S5 for details).

### Quantification of donor contributions to outcome communities following microbial community coalescence

**Figure 3:**
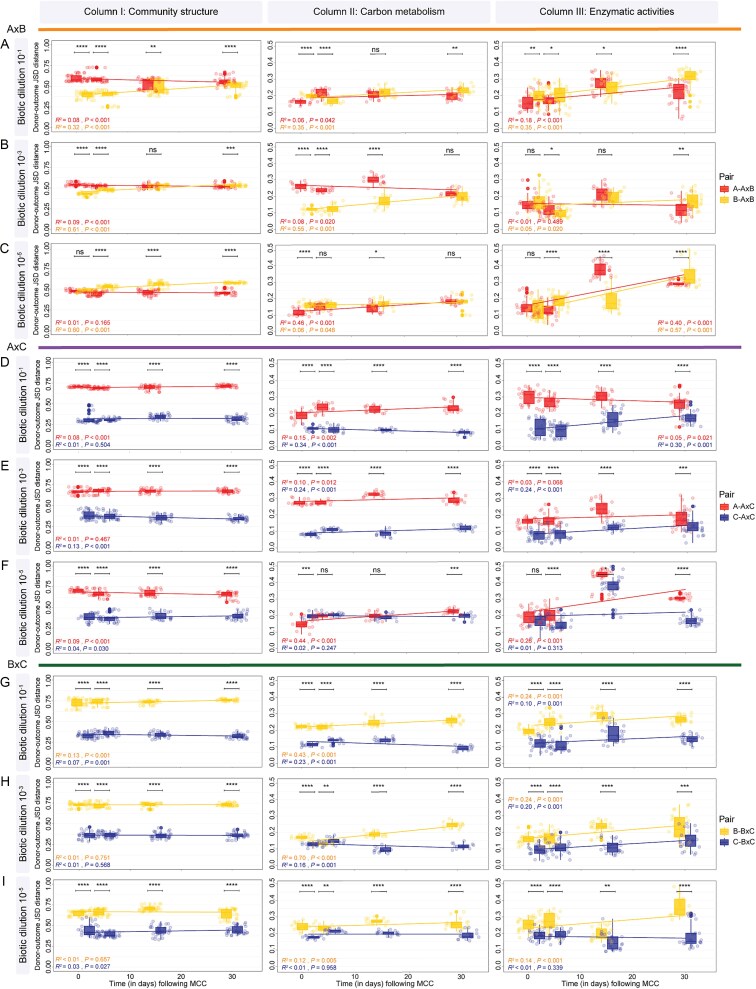
Boxplots and linear regressions displaying the relative contribution of donors to the outcome community structure (Column I), carbon metabolic profile (Column II), and enzymatic activities (Column III) over time based on donor–outcome JSD distances (A) AxB at the biotic dilution 10^−1^, (B) AxB at the biotic dilution 10^−3^, (C) AxB at the biotic dilution 10^−5^, (D) AxC at the biotic dilution 10^−1^, (E) AxC at the biotic dilution 10^−3^, (F) AxC at the biotic dilution 10^−5^, (G) BxC at the biotic dilution 10^−1^, (H) BxC at the biotic dilution 10^−3^, and (I) BxC at the biotic dilution 10^−5^. Statistical differences in donor relative contribution to the outcome community were determined using nonparametric Mann–Whitney *U* tests and are denoted by an asterisk above the boxplot (“ns,” *P* > .05; ^*^P < .05; ^*^^*^P < .01; ^*^^*^^*^P < .001; ^*^^*^^*^^*^P < .0001). Lower distances represent a greater contribution of the donor to the outcome community (i.e. dominance).

We found the relative contribution of donor communities to the outcome bacterial community to vary over time following MCC. In some cases, the dominance of donors shifted over time or was found to display distinct patterns when considering the biotic dilution treatments (i.e. 10^−1^ vs. 10^−5^) (Fig. 3). Specifically, JSD distances of treatment AxB^−1^ revealed a higher contribution of Donor B^−1^ throughout the experiment (*P <* .01), although differences in donor relative contributions reduced over time. For the treatment set AxB^−3^, a higher contribution of Donor B^−3^ was found at Days 1 and 5 (*P* < .0001), followed by a statistically equal contribution of donors A^−3^ and B^−3^ at Day 15 (*P* > .05), and finally shift to a higher contribution of Donor A^−3^ at Day 30 (*P* < .001). The treatment set AxB^−5^, however, showed equal contributions of both donors at Day 1 (*P* > .05), followed by a higher contribution of Donor A^−5^ on Days 5, 15, and 30 (*P* < .0001). Overall, the JSD distance of Donor A*^x^* to the outcome community showed a negative trend over time (e.g. negative slopes), representing a progressive increase in the contribution of this donor community over the sampling period (*P* < .001), except for A^−5^ with no correlation between JSD distance of Donor A^−5^—outcome community and time (*P* > .05). In contrast, Donor B*^x^* showed a positive trend in JSD distances over time, representing a temporal decrease in the contribution of this donor in the treatment sets AxB*^x^* (*P* < .001). For treatments AxC*^x^* and BxC*^x^*, Donor C*^x^* showed a greater contribution across all time points and dilutions (*P* < .0001) (Fig. 3D–I [Column I]). Linear modeling of the JSD distances between donor communities and the outcome communities revealed a temporal decrease in the contribution of A^−1^ to AxC^−1^, B^−1^ to BxC^−1^, and an increase in the contribution of A^−5^ to AxC^−5^ (*P* < .001). In contrast, Donor C*^x^* showed a temporal increase in its contribution to the outcome community in treatments AxC^−3^ and BxC^−1^, and a decrease in AxC^−5^ and BxC^−5^ over time (*P* < .05) (see Supplementary Results and Supplementary Table S6 for details).

This quantitative modeling approach was also applied to understand shifts in bacterial community carbon metabolic profiles (e.g. Biolog Ecoplate™ assays). Similarly, our results revealed distinct patterns in the contribution of the donors to the outcome metabolic profile over time (Fig. 3 [Column II]). Specifically, for the treatment AxB^−1^, Donor A^−1^ had a higher contribution at Day 1 A^−1^ (*P* < .0001), followed by a shift to the dominance of Donor B^−1^ (*P* < .0001) at Day 5. At Day 15, we observed statistically similar contributions from both donors (*P* > .05), followed by a higher contribution of Donor A^−1^ at Day 30 (*P* < .01). This pattern differed for the treatment AxB^−3^, which showed a higher contribution of Donor B^−3^ at Days 1, 5, and 15 (*P* < .0001), and an equal contribution from both donors at Day 30 (*P* > .05). However, for the treatment AxB^−5^, Donor A^−5^ had a higher contribution at Days 1 and 15 (*P* < .0001), although statistically equal contributions of both donors at Days 5 and 30 (*P* > .05) (Fig. 3A–C [Column II]). In the treatment sets AxC*^x^* and BxC*^x^*, Donor C*^x^* showed a greater contribution to the outcome metabolic profile across most sampling times and dilution treatments (*P* < .01), except at AxC^−5^ and BxC^−3^. Despite Donor A^−5^ showed a higher contribution to the outcome metabolic profile of AxC^−5^ at Day 1 (*P* < .001), this was followed by a statistically equal contribution of both donors at Days 5 and 15 (*P* > .05), and a higher contribution of Donor C^−5^ on Day 30 (*P* < .001) (Fig. 3F [Column II]). Lastly, for the treatment BxC^−3^, Donor C^−3^ had a higher contribution on Days 1, 15, and 30 (*P* < .0001), but Donor B^−3^ showed a higher contribution on Day 5 (*P* < .01) (see Supplementary Results and Supplementary Table S7 for details).

As for extracellular enzymatic activities, shifts in the relative contribution of donors were also observed (Fig. 3 [Column III]). For the treatment set AxB^−1^, a higher contribution of Donor A^−1^ was found at Days 1, 5, and 30 (*P* < .05), whereas Donor B^−1^ had a higher contribution at Day 15 (*P* < .05). Statistically equal contributions were found in treatment AxB^−3^ at Days 1 and 15 and AxB^−5^ at Day 1 (*P* > .05). However, Donor B^−3^ had a higher contribution to enzymatic activities in the outcome of AxB^−3^ at Day 5 (*P* < .05), and Donor A^−3^ was dominant at Day 30 (*P* < .01). For the treatment AxB^−5^, Donor A^−5^ showed a higher contribution at Days 5 and 30 (*P* < .0001), and Donor B^−5^ was dominant at Day 15 (*P* < .0001). However, the donor contributions to outcome enzymatic activities of treatments AxC*^x^* and BxC*^x^* showed a greater contribution of Donor C*^x^* throughout the treatments and across time points (*P* < .05), except on AxC^−5^ at Day 1, which had equal contributions of the donors (*P* > .05) (see Supplementary Results and Supplementary Table S8 for details).

### Correlations between donor contributions to community structure and functioning

**Figure 4:**
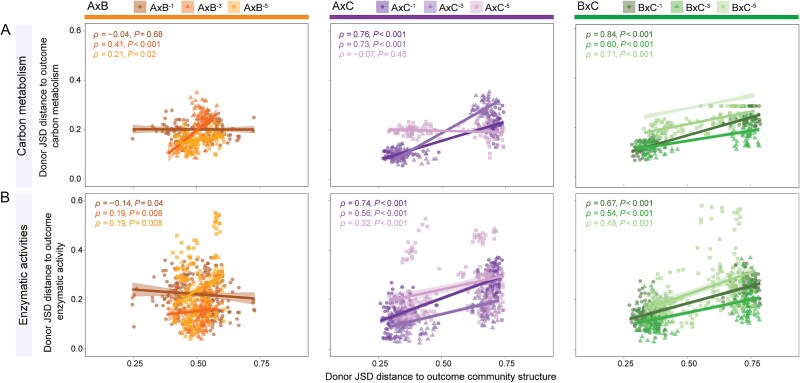
Scatterplots depicting the relationship between the contribution of the donor communities to the outcome community structure and their contribution to the (A) outcome community carbon metabolism and (B) outcome community enzymatic activities.

Correlational analyses were used to determine whether donor dominance in community structure is associated with donor dominance in community functioning (i.e. carbon metabolisms and enzymatic activities). In general, our results revealed significant positive correlations between the contribution of donor communities to outcome community structure and their contribution to carbon metabolic profiles for the treatments AxB^−3^ (*ρ* = 0.41, *P* < .001), AxB^−5^ (*ρ* = 0.21, *P* < .05), AxC^−1^ (*ρ* = 0.76, *P* < .001), AxC^−3^ (*ρ* = 0.73, *P* < .001), BxC^−1^ (*ρ* = 0.84, *P* < .001), BxC^−3^ (*ρ* = 0.60, *P* < .001), and BxC^−5^ (*ρ* = 0.71, *P* < .001) (Fig. 4A). However, no significant correlation was found for the treatment AxB^−1^ (*P* = .68) and AxC^−5^ (*P* = .45). Similarly, significant positive correlations between the contribution of donor communities to outcome community structure and their contribution to enzymatic activities were found for the treatments AxB^−3^ (*ρ* = 0.19, *P* < .01), AxB^−5^ (*ρ* = 0.19, *P* < .01), AxC^−1^ (*ρ* = 0.74, *P* < .001), AxC^−3^ (*ρ* = 0.56, *P* < .001), AxC^−5^ (*ρ* = 0.32, *P* = .001), BxC^−1^ (*ρ* = 0.67, *P* < .001), BxC^−3^ (*ρ* = 0.54, *P* < .001), and BxC^−5^ (*ρ* = 0.45, *P* < .001). The exception was the treatment AxB^−1^, which displayed a negative correlation (*ρ* = −0.14, *P* < .05) (Fig. 4B).

### Quantifying the importance of abiotic variables modulating biotic community coalescence

**Figure 5:**
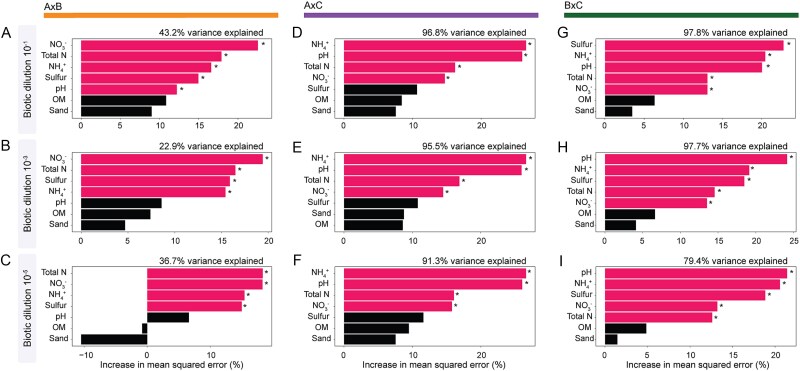
Bar plots displaying the individual and cumulative variance explained by abiotic variables in shifts in community structure following MCC. Treatment set AxB at the biotic dilutions (A) 10^−1^, (B) 10^−3^, (C) 10^−5^, treatment AxC at the biotic dilutions (D) 10^−1^, (E) 10^−3^, (F) 10^−5^, and treatment BxC at the biotic dilutions (G) 10^−1^, (H) 10^−3^, (I) 10^−5^. The importance of each abiotic factor is represented by the percentage of increase in mean squared error. Statistically significant factors are denoted with asterisks next to red bars (^*^P < .05).

To determine the extent to which changes in abiotic conditions after MCC contribute to shifts in community structure, we performed RF regression using the absolute delta variation (e.g. the absolute difference in the value between donor and outcome environments) of individual abiotic factors (e.g. sand, pH, OM, N-NH_4_^+^, N-NO_3_^−^, TN, and S) and the pairwise Bray–Curtis dissimilarities between donors and mixtures (Fig. 1C). Our modeling results revealed variations in soil pH, N-NH_4_^+^, N-NO_3_^−^, and TN pools to be the most important factors predicting shifts in community structure across all the treatment sets (*P* < .05) (Fig. 5 and Supplementary Table S9). In addition, shifts in S were found to be a significant factor for determining community change in treatments AxB*^x^* and BxC*^x^*. Variation in soil pH was shown to affect the outcome communities in the treatments AxB^−1^, AxC*^x^*, and BxC*^x^* (*P* < .05). It is important to notice that the total variance explained varied across treatment sets and biotic dilutions, with overall lower percentages in the treatment set AxB*^x^*. Specifically, our RF models explained 43.2% of the variance in the treatment AxB^−1^, 22.9% in the treatment AxB^−3^, and 36.7% in the treatment AxB^−5^. By contrast, RF analysis revealed that treatments including the Donor C (i.e. AxC*^x^*, BxC*^x^*) explained >90% of community variations over time (i.e. AxC*^x^*: 96.8% for AxC^−1^, 95.5% for AxC^−3^, 91.3% for AxC^−5^, BxC*^x^*: 97.8% for BxC^−1^, 97.7% for BxC^−3^), except for the treatment BxC^−5^ (with 79.4% of variance explained) (Fig. 5).

## Discussion

### Disentangling the ecological processes structuring community reassembly following microbial community coalescence

Ecological communities are structured through the dynamic interplay of four high-level processes, varying in their relative contribution over time (i.e. selection, ecological drift, dispersal, and diversification—*sensu* [32]). Within our experimental system, it is plausible to assume that dispersal was restricted to the initial mixing of distinct ecological communities (i.e. at Day 0), and that diversification had negligible effects on species distributions given the short experimental timeframe (i.e. 30 days). Therefore, community reassembly following experimental MCC was largely determined by selection (e.g. biotic and abiotic) and, to a lesser extent, ecological drift. The latter is known to have a stronger effect on species that occur at low abundances (see [32–34]). Besides, it is worth noting that our microcosm experiment was designed to minimize the effect of drift on community reassembly (e.g. homogenization of multiple inoculum bags and the construction of several experimental replicates). In this way, it is plausible to assume that selection was the dominant process structuring community reassembly in our system.

Ecological selection operates through two distinct lower level processes: (i) abiotic filtering (i.e. abiotic factors imposing “environmental filtering”) and (ii) biotic filtering (i.e. species interactions resulting in “niche partitioning”). Disentangling the relative importance of these often-intertwined low-level processes remains a challenge. This mostly occurs because (i) MCC across divergent systems varies in the relative proportions at which the abiotic and biotic parts are mixed (see [1] for details), and (ii) biotic species interactions can also exert an effect on abiotic properties following MCC [6]. With that, the donors’ contribution to the outcome community/abiotic properties ranges from symmetric (i.e. donors equally contribute to properties—species/functions/abiotic characteristics; no dominance) to asymmetric (i.e. the disproportional contribution of donors to the outcome community; dominance) (see [35]). As the balance of donor dominance is primarily affected by the ratios of mixing [1], we intentionally mixed samples in each treatment at a 50:50 ratio. This allowed us to confront the null hypothesis where, in the absence of variation in species fitness, ecological interactions, and dominant abiotic filtering, MCC would result in an outcome community composed of equal parts of the donors (i.e. symmetric with no dominance) [1, 35].

In contrast to this null expectation, our results revealed strong patterns of abiotic and biotic donor dominance in the treatment sets AxC*^x^* and BxC*^x^* across all biotic dilution and time points (Figs 2H and I and 3D–I [Columns I–III] and 5). This was largely determined by Donor C imposing abiotic dominance (i.e. asymmetric abiotic outcome), where environmental filtering disproportionally favored the establishment of taxa pre-adapted to Donor C’s abiotic conditions (i.e. biotic asymmetric outcome)—a phenomenon also known as “home-field advantage” [36, 37]. Despite our experimental system being based on only three distinct soils, this effect was similarly observed in a previous study of MCC in aquatic systems. In brief, the authors showed that the mixing of freshwater and marine ecosystems resulted in the persistence of elevated levels of salinity as a stringent environmental filter, which disproportionally favored the engraftment of microbial taxa originally from the marine system [38]. Particularly in our system, Soil C displayed higher contents of clay, OM, higher pH, and CEC (see Supplementary Tables S2 and S3). Hence, it is likely that these physicochemical properties provided a buffering capacity to Soil C when subjected to MCC with Soils A or B, thus resulting in greater donor abiotic and biotic dominance over time. Additionally, the existence of this upfront abiotic environmental filter in treatment sets AxC*^x^* and BxC*^x^* (Fig. 5) is supported by the lack of temporal dynamics in community structure dominance across all biotic dilutions, where Donor C dominated from Day 1 to the end of the experiment (i.e. Day 30), and shifting abiotic properties after MCC explained >90% of the variation structure in these treatment sets (Fig. 5D–I). Specifically, RF modeling showed patterns of community reassembly following MCC in the treatment sets AxC*^x^* and BxC*^x^* to be largely determined by shifts in soil pH and nitrogen pools (i.e. N-NH_4_^+^, TN, and NO_3_^−^), all of which are well-known to impose distinct levels of ecological selection on soil bacteria [39–42]. Despite methodological challenges associated with partitioning the relative influences of abiotic and biotic filters in our system, these results collectively provide support for the notion that abiotic environmental filtering exerts an upfront selection on microbial community reassembly following MCC, thus favoring species adapted to abiotic conditions following MCC (see Hypotheses i and iii).

Our results also revealed a contrasting pattern where, in the absence of stringent environmental filtering and “home-field advantage,” species biotic interactions become more important for determining the temporal dynamics of community reassembly following MCC. Specifically, in the treatment set AxB*^x^*, MCC resulted in a more symmetric abiotic outcome (as compared to the treatment sets AxC*^x^* and BxC*^x^*), with a slight dominance of Donor A*^x^* (Fig. 2G [Column I]). In this case, the edaphic conditions of the outcome system imposed approximately equal environmental selection pressure on taxa from both donor communities (see Supplementary Tables S2 and S3). This is evidenced by shifts in abiotic variables in the treatment set AxB*^x^* explaining <45% of the community turnover following MCC (Fig. 5A–C), suggesting a decreased importance of environmental filtering relative to the AxC*^x^* and BxC*^x^* treatment sets. Moreover, in the treatment sets AxB*^x^*, we observed contrasting patterns in donor community dominance across the biotic dilution gradient over time. For example, whereas in treatments AxB^−1^ and AxB^−3^, donor’s contributions converged to a symmetric outcome (~0.50 JSD distances at Day 30; Fig. 3A and B [Column I]), in the treatment AxB^−5^, a divergent pattern in donor contributions was observed, indicating temporal dynamics in donor dominance. This was likely driven by variations in biotic interactions over time and treatment dilutions, which become more important in determining patterns of species engraftment in the absence of a stringent upfront abiotic filter [43, 44] (Fig. 3C [Column I]). Moreover, these results also point to the importance of differences in species cohesiveness within donor communities (i.e. metabolic interdependences of two or more species) that are disrupted during experimental community dilution [16, 45]. That is, the serial biotic dilution treatments in our microcosm randomly disrupted ecological interactions and cohesiveness within donor communities (see also [46]). This is likely the cause of the observed dynamic temporal patterns in donor contributions to outcome communities across the treatment sets AxB*^x^* across the distinct dilution sets (Fig. 3A–C [Column I]). Collectively, these results corroborate our initial Hypothesis *ii*, stating that biotic community dilution may exert an effect on donor contributions to the outcome communities due to a reduction in community complexity.

### Exploring the impacts of microbial community coalescence on community functioning

Using a combination of two community functioning assays (i.e. carbon metabolic profiling and enzymatic activities), our results revealed contrasting patterns in the relationship between shifts in community structure and function following MCC (Fig. 4). These results largely align with our interpretation of the interplay of abiotic and biotic filtering and the understanding of functional similarity relationships in biologically diverse microbial communities [47]. In brief, the most common outcome revealed significantly positive correlations between shifts in donor contributions to community structure and functional profiles following MCC (Fig. 4). These trends were mostly apparent in the treatment sets AxC*^x^* and BxC*^x^* (except for the metabolic profile at the treatment AxC^−5^), where abiotic dominance led to a greater contribution of Donor C*^x^* to the outcome community (Fig. 4, Column 3). In this case, it is plausible to assume that upfront abiotic selection steers community structure by favoring the engraftment from one of the donor communities, and this is directly reflected in the functionality of the outcome community (i.e. home-field advantage) [48]. In contrast, in the absence of an upfront stringent abiotic filter, we found that the level of community biotic dilution determines the relationship between shifts in community structure and functioning following MCC. First, at a low level of community dilution (i.e. treatment AxB^−1^), no significant correlation was observed between shifts in community structure and metabolic profile, and a marginally significant negative correlation was found between shifts in community structure and enzymatic activities. This may indicate that the high level of functional similarity among taxa in both donor communities decouples the relationship between community structure and functioning—in this case, due to similar environments hosting similar taxa. That is, even if biotic interactions favor the disproportionate engraftment of taxa from one of the donors, these variations may be insufficient for determining functional divergence in the outcome community, as most of these functions are redundantly present in taxa from both donor communities. Second, at higher biotic dilution treatments (i.e. treatment sets AxB^−3^ and AxB^−5^), it becomes apparent that the failure of specific taxa to engraft can elicit a direct effect on community functioning due to the reduction of functional redundancy across taxa from both donor communities—an artifact of the dilution treatment. This results in a coupled relationship between shifts in community structure and functioning following MCC. It is worth noting that distinct functions vary in terms of redundancy across multiple microbial taxa; however, most of the functions measured in this study (e.g. carbon utilization and enzymatic activities) are known to be performed by multiple soil organisms (also see [47, 49]). Collectively, these results partially support our initial hypothesis of coupled shifts in community structure and functioning following MCC. We also partially countered this expectation by showing that functional redundancy can decouple this relationship, especially in the absence of an upfront stringent abiotic filter responsible for determining community reassembly following MCC.

### Advantages and limitations

Several metrics and approaches have been employed to study MCC across systems (e.g. SourceTracker [50], FEAST (Fast expectation-maximization for microbial source tracking) [51], RECAST (Recipient intestine colonization analysis tool) [52], reviewed in detail in [6]). These methods focus on detecting the engraftment of individual species or strains within a system after MCC. However, a common limitation of these approaches is that their precision tends to diminish as microbial community diversity increases due to redundancy in taxa between systems. To address this issue, we developed a beta-diversity–based framework that quantifies the relative contribution of donor communities to the outcome community through time-series analysis. This versatile framework can be applied to various multivariate dataset types, including amplicon sequencing, metagenomics, functional profiles, and abiotic variable measurements. In addition, unlike traditional methods that dissect individual species or strain engraftment, our approach evaluates community-level properties by using community distances to measure the similarity between donor and outcome communities. This aligns with recent research efforts on the theme of invasion biology, in which species invasiveness is largely influenced by community emergent properties (e.g. resilience, resistance, resource utilization) rather than by specific species or functions depicted at the individual species level [53–55]. Last, we also acknowledge this approach is not without limitations. First, the detection of species/strain-specific engraftment might be important and relevant in some systems (e.g. clinical interventions, see [56]), and this method does not allow for the determination of strain- or species-level engraftment dynamics. Second, the use of different distance metrics may produce inconsistent results, though the general patterns lead to consistent conclusions (Supplementary Figs S6–S8). Despite these minor drawbacks, we believe adopting this framework provides a robust and scalable tool for studying MCC, particularly in complex and dynamic microbial systems. This approach can be broadly implemented and interpreted in line with the beta-diversity metrics commonly employed in microbiome sciences and community ecology.

## Supplementary Material

Supplementary_Information_Bresciani_et_al_2025_wraf041

## Data Availability

Raw sequence reads obtained in this study were deposited into NCBI Sequence Read Archive (SRA) database (BioProject: PRJNA1147197). The rarefied sequencing data and R scripts used in this study are available at GitHub (https://github.com/luanabresciani/MCC-Soil-experiment). The scripts of the beta-diversity quantitative model used to quantify the relative contribution of donors to the outcome community are available at GitHub (https://github.com/KoslickiLab/Beta-diversity-of-mixtures-over-time).
