# Supplementary material for: Interplay of ecological processes modulates microbial community reassembly following coalescence
Source: ISME J. 2025 Apr 3;19(1):wraf041. doi: 10.1093/ismejo/wraf041 (PMC11971568; doi:10.1093/ismejo/wraf041)
Supplement: Supplementary_Information_Bresciani_et_al_2025_wraf041 [file supplementary_information_bresciani_et_al_2025_wraf041.pdf]

SUPPLEMENTARY INFORMATION

Interplay of ecological processes modulates microbial community reassembly following coalescence

Authors

Luana Bresciani[1,2,3], Gordon F Custer[1,2,3,4], David Koslicki[2,3,5,6], Francisco Dini-Andreote[1,2,3*]

Affiliations

[1]Department of Plant Science, The Pennsylvania State University, University Park, PA, USA

[2]Huck Institutes of the Life Sciences, The Pennsylvania State University, University Park, PA, USA

[3]One Health Microbiome Center, The Pennsylvania State University, University Park, PA, USA

[4]Department of Natural Sciences, The University of Maryland Eastern Shore, Princess Anne, MD, USA

[5]Department of Computer Science and Engineering, The Pennsylvania State University, University Park, PA, USA

[6]Department of Biology, The Pennsylvania State University, University Park, PA, USA

***Corresponding author:** Francisco Dini-Andreote, Department of Plant Science & Huck Institutes of the Life Sciences, The Pennsylvania State University, University Park, State College, PA, 16802, USA. E-mail: andreote@psu.edu, phone: +1 814 863 2188.

The Supplementary Information includes Supplementary Methods, Supplementary Results, Supplementary Figures S1–S8, and Supplementary Tables S1–S9.

Supplementary Methods

Soil physicochemical analysis

Soil physical and chemical analyses were performed at Penn State's Agricultural Analytical Services Laboratory. Soil physical analyses were performed on soil samples before gamma irradiation as these properties are not expected to change following irradiation. These included the determination of soil particle sizes (e.g., clay, sand, and silt content) via the hydrometer method [1] and sand sieve (i.e., gravel, very coarse sand, coarse sand, medium sand, fine sand, very fine sand, and fines) using US Standard Sieve numbers: 10 (> 2 mm), 18 (2.0 – 1.0 mm), 35 (1.0 – 0.5 mm), 60 (0.5 – 0.25 mm), 140 (0.25 – 0.1 mm), and 270 (0.1 – 0.05 m), respectively.

Soil chemical analyses were performed on soil samples before and after gamma irradiation and on mixtures of soil pairs in equal proportions (in line with our MCC experiment, see below) after gamma irradiation. For a detailed overview of soil chemical properties before and after gamma irradiation, see **Supplementary Table S1**. Chemical analyses included the determination of soil pH, potential acidity, phosphorus (P), potassium (K), calcium (Ca), magnesium (Mg), zinc (Zn), copper (Cu), sulfur (S), cation exchange capacity (CEC), organic matter (OM), total carbon (TC), soluble salts, nitrate ($N\text{-}NO_3^-$), ammonium ($N\text{-}NH_4^+$), and total nitrogen (TN). Briefly, soil pH was measured in a 1:1 (*v/v*) soil-deionized water slurry with standard electrodes [2]. Exchangeable acidity was measured using the same soil-water slurry used for pH determination with 5 ml modified Mehlich buffer solution added [3]. Extractable P, K, Ca, Mg, Cu, Zn, and S were determined using 2.5 g of air-dried soil and 25 ml of Mehlich 3 extracting solution [4]. Soil CEC was estimated based on extractable K, Ca, and Mg [5]. Soil OM was determined by weight loss on ignition [6], and total C by combustion [7]. Soluble salts were measured by 1:2 (v/v) soil: water extract method using 10 cm^3 of dried and sieved soil with 20 ml of de-ionized water and 0.01 N KCl standard solution for electrical conductivity measurements [8]. Soil $N\text{-}NO_3^-$ was colorimetrically measured in a solution using the cadmium reduction method [9], and $N\text{-}NH_4^+$ was determined using the diffusion-conductivity technique on 5 g of air-dried soil with 25 ml of 2 N KCl extraction reagent [10]. The total N was measured by combustion of air-dried soil [11, 12].

Extracellular enzymatic activity

Extracellular enzymatic activities were determined for β-glucosidase (BG), β-xylosidase (BX), α-glucosidase (AG), cellobiohydrolase (CBH), *N*-acetyl-β-glucosaminidase (NAG), leucine aminopeptidase (LAP), acid phosphatase (PHOS), and sulfatase (SUL). First, a preliminary assay was conducted to ensure nonlimiting substrate availability and maximum potential of enzymatic activity (V_{max}) [13]. This preliminary assay consisted of four substrate concentrations (i.e., 200, 1000, 2000, and 4000 μM), and activities were determined at five time points (i.e., 1, 2, 4, 6, 8, and 10 hours). The preliminary assay was performed on the three control treatments, representing the expected variability in enzymatic activity across samples. Briefly, 1g of soil sample was homogenized using 100 ml of pH-adjusted sodium acetate buffer [50 (pH 5.8)] for 30 s using a Magic Bullet Blender. Buffer pH was chosen to minimize the differences between the buffer and the soil pH. For the enzyme assays, 200 μL of soil slurry was added to 50 μL of substrate in a 96-well microplate and incubated at 20 °C for 10 hrs. Fluorescence of each soil sample was measured using four technical replicates. Hydrolytic enzyme activity was assessed by sample fluorescence read at 360 nm excitation wavelength and 450 nm emission wavelength, using a BioTeck Synergy H1 Microplate reader (Agilent BioTechnologies, CA, USA). Negative controls (sample without substrate addition) were used to correct for background fluorescence values. Additionally, a quench control (sample homogenate with standards) was used to correct for soil particle interference. The standard 10 μM of 4-methylumbelliferone for BG, BX, AG, CBH, NAG, PHOS, and SUL and 7-amido-4-methylcoumarin hydrochloride was used for LAP. Enzymatic activities were calculated and reported as nmol or μmol of substrate converted per hour per gram of dry soil (nmol/μmol h^{-1} g^{-1}) [14]. The preliminary assay was used to determine the highest

average enzyme activity across incubation times and substrate concentrations. Then, a single substrate concentration and incubation time were selected to be used for each substrate and across all samples. The final enzyme assays were performed following the same protocol using the substrate concentration and incubation time that produced the maximum potential of enzymatic activity (V_{max}).

Supplementary Results

Temporal effect of the relative contribution of donors to the outcome community following MCC

Temporal effect of donors' contributions to the outcome community were evaluated using linear regression models (LM). The JSD distances of A^{-1} – AxB^{-1} and A^{-3} – AxB^{-3} presented a slight negative correlation with time (slope = -0.001, $P < 0.001$ A^{-1} – AxB^{-1}; slope =-0.001, $P < 0.001$ A^{-3} – AxB^{-3}), but no significant correlation of A^{-5} – AxB^{-5} distances was found with time ($P > 0.05$). However, the distances of B^{-1} – AxB^{-1}, B^{-3} – AxB^{-3} and B^{-5} – AxB^{-5} showed positive correlation with time, as decreasing donors contribution over time (slope = 0.004, $P < 0.001$ B^{-1} – AxB^{-1}; slope = 0.003, $P < 0.0001$ for B^{-3} – AxB^{-3}, and slope = 0.003, $P < 0.0001$ B^{-5} – AxB^{-5}). For treatment AxC^{x}, distances of A^{-1} – AxC^{-1} and A^{-5} – AxC^{-5}, presented positive and negative correlation with time, respectively (slope = 0.001, $P < 0.001$ A^{-1} – AxC^{-1}; slope = -0.001, $P < 0.001$ A^{-5} – AxC^{-5}), whereas no significant correlation of A^{-3} – AxC^{-3} distance with time ($P > 0.05$). No significant correlation was found with time and distance C^{-1} – AxC^{-1}, but negative correlation was observed on distances C^{-3} – AxC^{-3} and time (slope = -0.001, $P < 0.0001$) and positive correlation on C^{-5} – AxC^{-5} with time (slope = 0.001, $P = 0.03$). Distance of B^{-1} – BxC^{-1} presented positive correlation with time (slope = 0.001, $P < 0.0001$), whereas B^{-3} – BxC^{-3} and B^{-5} – BxC^{-5} distances did not present correlations with time ($P > 0.05$).Lastly, the distances of donors C^{-1} – BxC^{-1} presented negative correlation with time (slope = -0.001, $P < 0.01$), and C^{-5}– BxC^{-5} distance showed a slight positive correlation (LM, slope = 0.001, $P = 0.03$). Distance of C^{-3} – BxC^{-3} showed no significant correlation with time ($P > 0.05$).

The JSD distances of A^{-1} – AxB^{-1} and A^{-5} – AxB^{-5} presented slight positive correlation with time (slope =0.001, $P = 0.04$ A^{-1} – AxB^{-1}; slope = 0.002, $P < 0.0001$ A^{-5} – AxB^{-5}), whereas A^{-5} – AxB^{-3} showed negative correlation with time following coalescence (slope = -0.001, $P = 0.02$). Distances of B^{-1} – AxB^{-1}, B^{-3} – AxB^{-3} and B^{-5} – AxB^{-5} presented positive correlation with time (slope = 0.002, $P < 0.0001$ B^{-1} – AxB^{-1}; slope = 0.003, $P < 0.0001$ B^{-3} – AxB^{-3}; and slope = 0.001, $P < 0.0001$ B^{-5} – AxB^{-5}). For community AxC^{x}, distances A^{-1} – AxC^{-1}, A^{-3} –AxC^{-3} and A^{-5}–AxC^{-5} showed positive correlation with time (slope = 0.001, $P = 0.002$ A^{-1} – AxC^{-1}; slope = 0.001, $P = 0.01$ A^{-3} – AxC^{-3}; and slope = 0.002, $P < 0.0001$ A^{-5} – AxC^{-5}). To the other side, the distances C^{-1} – AxC^{-1} presented a negative correlation with time (slope = -0.001, $P < 0.0001$), C^{-3}– AxC^{-3} distances presented a positive correlation (slope= 0.001, $P < 0.0001$), and C^{-5}– AxC^{-5} showed no significant correlation ($P > 0.05$). Distances B^{-1} – BxC^{-1}, B^{-3} – BxC^{-3} and B^{-5}– BxC^{-5} presented positive correlation with time (slope = 0.001, $P < 0.0001$ B^{-1} – BxC^{-1}; slope = 0.003, $P < 0.0001$ B^{-3} – BxC^{-3}; and slope = 0.001, $P < 0.01$ B^{-5} – BxC^{-5}). For distances of C^{-1} – BxC^{-1} and C^{-3} – BxC^{-3} presented a negative correlation with time (slope = -0.001, $P < 0.0001$ C^{-1} – BxC^{-1}; slope = -0.001, $P < 0.01$ C^{-3} – BxC^{-3}), whereas C^{-5}– BxC^{-5} showed no correlation with time ($P > 0.05$).

Correlational analysis between A^{-1} – AxB^{-1} and A^{-5} – AxB^{-5} distances on enzymatic functioning were positive with time following coalescence (slope = 0.003, $P < 0.0001$ A^{-1} – AxB^{-1}; slope = 0.006, $P < 0.0001$ A^{-5} – AxB^{-5}), while no correlation found for donor A^{-3} distances to coalesced community functioning ($P > 0.05$). Distances B^{-1} – AxB^{-1}, B^{-3} – AxB^{-3} and B^{-5} – AxB^{-5} presented positive correlation with time (slope = 0.004, $P < 0.0001$ B^{-1} – AxB^{-1}; slope = 0.001, $P = 0.02$ B^{-3} – AxB^{-3}, slope = 0.01, $P < 0.0001$ B^{-5} – AxB^{-5}). Distances A^{-1} – AxC^{-1} presented a negative correlation with time (slope = -0.001, $P = 0.02$), whereas A^{-3} – AxB^{-3} distances presented no significant correlation ($P > 0.05$) and A^{-5} – AxC^{-5} showed negative correlation with time following coalescence (slope = 0.005, $P < 0.0001$). On the other hand, distances of C^{-1} – AxC^{-1} and C^{-3} – AxC^{-3} presented a positive correlation with time following coalescence (slope = 0.003, $P < 0.0001$ C^{-1} – AxC^{-1}; slope = 0.002, $P < 0.0001$ C^{-3} – AxC^{-3}), and no correlation was found between distances of donor C^{-5} to coalesced AxC^{-5} and time ($P > 0.05$). For communities BxC^{x}, distances B^{-1} – BxC^{-1}, B^{-3} – BxC^{-3}, and B^{-5} – BxC^{-5} presented positive correlation with time following coalescence (slope = 0.002, $P < 0.0001$ B^{-1} – BxC^{-1}; slope = 0.003, $P < 0.0001$ for B^{-3} – BxC^{-3}, slope = 0.006, $P < 0.0001$ B^{-5} – BxC^{-5}). Distances C^{-1} – BxC^{-1} and C^{-3} – BxC^{-3} presented a positive

correlation with time (slope = 0.001, $P < 0.01$ C^{-1} – BxC^{-1}; slope = 0.002, $P < 0.0001$ C^{-3} – BxC^{-3}), and C^{-5} – BxC^{-5} distance presented no significant correlation with time ($P > 0.05$).

Supplementary Figures and Supplementary Tables

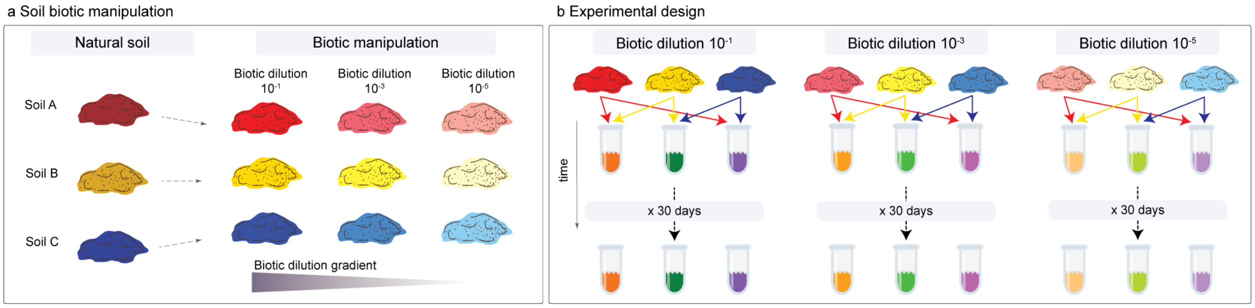

Supplementary Figure S1. Schematic illustration of the soil biotic manipulations and experimental design. (**a**) Natural soils A (red), B (yellow), and C (blue) were sterilized via gamma-irradiation (>35 kGy) and reinoculated at three levels of biotic dilution, using a dilution-to-extinction gradient (i.e., 10^{-1}, 10^{-3} and 10^{-5}). Biotic dilutions are represented by color intensity. (**b**) After biotic manipulations and microbial community stabilization for 60 days, MCC was performed by mixing the soils in pairs (i.e., 1:1 soil mixing) within the same biotic dilution treatment. The mixtures of A and B is represented in orange (e.g. yellow + red soils), A and C in green (e.g., yellow + blue soils), and B and C in purple (e.g., red + blue soils). The color intensity follows the biotic dilution treatments. The outcome community reassembly was investigated based on datasets of community structure, carbon metabolism, and enzymatic activities at day 0, 5, 15, and 30 after MCC.

Effects of soil sterilization on soil chemical properties

Soils A, B, and C before and after sterilization displayed small, albeit statistically significant differences in chemical properties. Soil pH, OM, P, Mg, Zn, acidity index, CEC, TC, and TN did not significantly change after sterilization. However, we observed a significant increase in N-NH_4^+ content for soils A, B and C after gamma-irradiation (P < 0.05). Similarly, soil A presented an increase in soluble salts after sterilization (P < 0.05), whereas soil B presented a decrease in Ca (P < 0.05), and soils B and C decreased in N-NO_3^- content (P < 0.05). Soil C showed a significant decrease in K (P < 0.05) and Cu (P < 0.05) and an increase in S after sterilization (P < 0.05) (**Supplementary Table S1**).

Supplementary Table S1. Chemical analysis of donor soils A, B, and C before (natural soils) and after sterilization (sterilized soil) by γ-irradiation. Values are means ± SD; n = 3. Means are followed by different superscripted lowercase letters (a-d) that represent statistically significant pairwise differences from each other at P < 0.05 (Tukey's HSD test). OM = organic matter; CEC = cation exchange capacity; TC = total carbon; TN = total nitrogen.

**Soil**	**pH** (H_2O)	**OM** %	**P** (ppm)	**K** (ppm)	**Mg** (ppm)	**Ca** (ppm)
Natural soil A	5.87 ± 0.09 ^{b}	0.770 ± 0.14 ^{c}	277.67 ± 32.0 ^{a}	61.33 ± 8.08 ^{c}	74.33 ± 5.03 ^{b}	469.17 ± 46.03 ^{b}
Sterilized soil A	5.80 ± 0.01 ^{b}	0.633 ± 0.02 ^{c}	294.33 ± 24.2 ^{a}	53.33 ± 4.16 ^{c}	71.33 ± 5.51 ^{bc}	375.40 ± 36.47 ^{bc}
Natural soil B	5.20 ± 0.07 ^{c}	1.690 ± 0.44 ^{b}	33.33 ± 2.52 ^{c}	33.33 ± 1.15 ^{d}	61.67 ± 7.37 ^{cd}	302.73 ± 56.26 ^{c}
Sterilized soil B	5.14 ± 0.03 ^{c}	1.457 ± 0.19 ^{b}	38.33 ± 1.53 ^{c}	29.67 ± 2.08 ^{d}	53.33 ± 2.52 ^{d}	193.97 ± 23.98 ^{d}
Natural soil C	6.82 ± 0.11 ^{a}	3.793 ± 0.10 ^{a}	101.67 ± 5.51 ^{b}	503.33 ± 25.5 ^{a}	501.33 ± 5.69 ^{a}	3798.73 ± 220.4 ^{a}
Sterilized soil C	6.77 ± 0.08 ^{a}	3.640 ± 0.07 ^{a}	122.00 ± 6.08 ^{b}	396.00 ± 16.5 ^{b}	481.33 ± 20.5 ^{a}	3493.63 ± 232.2 ^{a}

**Soil**	**Zn** (ppm)	**Cu** (ppm)	**S** (ppm)	**NO_3^-** (ppm)	**NH_4^+** (mg/kg)	**Soluble Salts** (mmhos/cm)
Natural soil A	12.43 ± 0.70 ^{b}	6.47 ± 0.45 ^{a}	12.07 ± 0.78 ^{d}	10.3 ± 1.26 ^{b}	0.888 ± 0.05 ^{e}	0.073 ± 0.01 ^{c}
Sterilized soil A	12.33 ± 1.31 ^{b}	6.17 ± 0.60 ^{a}	14.10 ± 1.35 ^{cd}	9.10 ± 0.35 ^{b}	4.460 ± 0.12 ^{c}	0.090 ± 0.00 ^{b}
Natural soil B	4.13 ± 1.07 ^{c}	1.33 ± 0.21 ^{d}	17.63 ± 0.45 ^{bc}	2.42 ± 0.08 ^{d}	4.513 ± 0.43 ^{c}	0.050 ± 0.00 ^{d}
Sterilized soil B	3.60 ± 0.17 ^{c}	1.00 ± 0.00 ^{d}	20.67 ± 1.06 ^{b}	1.03 ± 0.06 ^{e}	16.10 ± 0.62 ^{b}	0.077 ± 0.01 ^{bc}
Natural soil C	37.23 ± 2.71 ^{a}	4.77 ± 0.29 ^{b}	13.83 ± 2.05 ^{cd}	19.6 ± 0.28 ^{a}	2.672 ± 0.16 ^{d}	0.040 ± 0.00 ^{d}
Sterilized soil C	37.33 ± 2.36 ^{a}	3.57 ± 0.15 ^{c}	25.93 ± 2.58 ^{a}	3.13 ± 0.23 ^{c}	38.42 ± 1.16 ^{a}	0.313 ± 0.01 ^{a}

**Soil**	**Acidity index** (meq/100g)	**CEC** (meq/100g)	**TC** %	**TN** %
Natural soil A	2.60 ± 0.35 ^{b}	5.7 ± 0.36 ^{c}	0.723 ± 0.11 ^{c}	0.06 ± 0.01 ^{b}
Sterilized soil A	3.00 ± 0.35 ^{b}	5.6 ± 0.61 ^{c}	0.720 ± 0.00 ^{c}	0.06 ± 0.01 ^{b}
Natural soil B	6.90 ± 0.60 ^{a}	9.0 ± 0.35 ^{b}	1.173 ± 0.08 ^{b}	0.08 ± 0.01 ^{b}
Sterilized soil B	7.70 ± 0.35 ^{a}	9.2 ± 0.26 ^{b}	1.293 ± 0.01 ^{b}	0.08 ± 0.00 ^{b}
Natural soil C	1.33 ± 1.15 ^{b}	21.8 ± 1.04 ^{a}	2.540 ± 0.20 ^{a}	0.23 ± 0.01 ^{a}
Sterilized soil C	1.33 ± 1.15 ^{b}	21.3 ± 0.99 ^{a}	2.387 ± 0.05 ^{a}	0.22 ± 0.00 ^{a}

Effects of dilutions on soil bacterial diversity

Alpha diversity metrics were used to determine the effects of biotic dilutions on community diversity (see **Supplementary Figure S2 a-d**). Biotic dilutions are represented using the soil type followed by the superscripts $^{-1}$, $^{-3}$, and $^{-5}$ for dilutions 10^{-1}, 10^{-3}, and 10^{-5}, respectively. Soil A^{-1} presented a higher number of observed bacterial ASVs (742.5 ± 105.33), Chao1 index (747.36 ± 107.31), Shannon diversity index (5.51 ± 0.16), and evenness (0.84 ± 0.01) as compared to A^{-3} and A^{-5} ($P < 0.05$), whereas no significant difference was detected between A^{-3} and A^{-5} ($P > 0.05$). Likewise, soil B^{-1} showed a higher bacterial observed ASVs (473.83 ± 51.72), Chao1 index (478.61 ± 55.95) and Shannon index (5.21 ± 0.086), when compared to B^{-3} (412.83 ± 21.94 ASVs, 415.55 ± 24.48 Chao1, 4.76 ± 0.057 Shannon) ($P < 0.05$). However, no significant difference was detected between B^{-1} and B^{-5} (453.00 ± 20.94 observed ASVs, 454.97 ± 21.88 Chao1) ($P < 0.05$), except for in Shannon diversity (5.06 ± 0.046) ($P < 0.05$). A similar trend was found for evenness, with higher values observed in B^{-1} (0.85 ± 0.01), followed by B^{-5} (0.83 ± 0.01) and B^{-3} (0.79 ± 0.01) ($P < 0.05$). Last, soil C^{-1} had significantly higher observed ASVs (526.17 ± 15.01) and Chao1 index (529.83 ± 15.33) compared to C^{-3} and C^{-5} ($P < 0.05$). Shannon diversity values in C^{-1} (5.25 ± 0.09) and C^{-3} (5.15 ± 0.14) were statistically similar ($P > 0.05$), but higher than those in C^{-5} (4.78 ± 0.27) ($P < 0.05$). Evenness in C^{-1} was statistically similar to those in C^{-3} and C^{-5}, whereas the evenness in C^{-3} (0.86 ± 0.01) was significantly different from that in C^{-5} (0.81 ± 0.03) ($P < 0.05$).

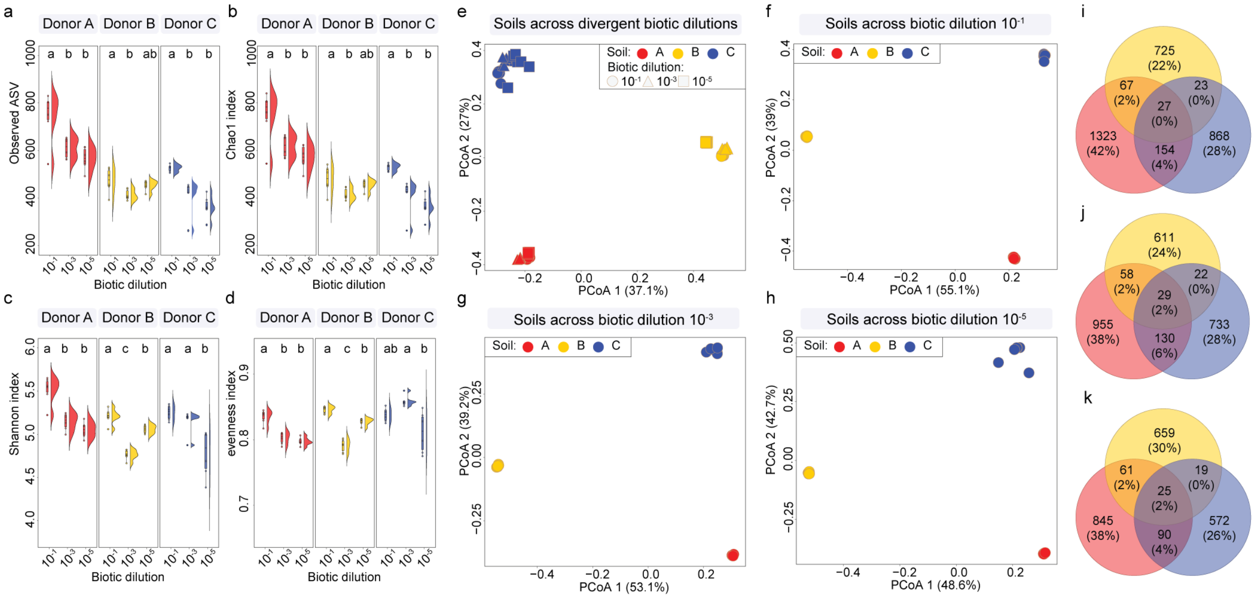

Supplementary Figure S2. Effects of biotic dilutions on community bacterial α-diversity. Richness estimations of (**a**) observed bacterial ASVs, (**b**) Chao1 index, (**c**) Shannon diversity index, and (**d**) evenness index are colored by soil type and different lowercase letters indicate significant differences at $P < 0.05$ (Tukey's HSD test) between biotic dilution treatments at day 1. Principal coordinate analysis (PCoA) based on Bray-Curtis distances shows the clustering of communities by (**e**) soil types and biotic dilution sets, (**f**) soil types across the biotic dilutions 10^{-1}, (**g**) soil types across the biotic dilutions 10^{-3}, and (**h**) soil types across the biotic dilutions 10^{-5}. Venn diagram depicting unique and shared ASVs between soils A, B, and C at day 1 on (**i**) biotic dilutions 10^{-1}, (**j**) biotic dilutions 10^{-3}, and (**k**) biotic dilutions 10^{-5}.

Soil physicochemical analysis

Supplementary Table S2. Values of soil physical properties of donors A, B, and C and outcome soils AxB, AxC, and BxC. Values are means ± SD; *n* = 3. Means are followed by different superscripted lowercase letters (a-f) that represents soil significant difference from each other at *P* < 0.05 (Tukey's HSD test).

**Soil**	**Sand**	**Silt**	**Clay**	**Gravel retained**	**Very coarse sand retained**
	%	%	%	%	%
A	91.26 ± 1.53 ^{a}	5.70 ± 0.87 ^{f}	3.03 ± 0.70 ^{e}	0.00 ± 0.00 ^{c}	3.367 ± 2.37 ^{a}
B	76.66 ± 1.36 ^{c}	16.30 ± 0.92 ^{d}	6.97 ± 0.65 ^{c}	0.00 ± 0.00 ^{c}	0.267 ± 0.06 ^{d}
C	15.86 ± 0.25 ^{f}	54.83 ± 0.67 ^{a}	29.33 ± 0.70 ^{a}	69.2 ± 1.37 ^{a}	0.800 ± 0.10 ^{bcd}
A x B	83.96 ± 1.33 ^{b}	11.00 ± 0.69 ^{e}	5.00 ± 0.68 ^{d}	0.00 ± 0.00 ^{c}	1.817 ± 1.20 ^{abc}
A x C	53.56 ± 0.85 ^{d}	30.27 ± 0.29 ^{c}	16.18 ± 0.70 ^{b}	34.6 ± 0.69 ^{b}	2.083 ± 1.19 ^{ab}
B x C	46.26 ± 0.68 ^{e}	35.57 ± 0.28 ^{b}	18.15 ± 0.68 ^{b}	34.6 ± 0.69 ^{b}	0.533 ± 0.03 ^{cd}

**Soil**	**Coarse sand retained**	**Medium sand retained**	**Fine sand retained**	**Very fine sand retained**	**Fines retained**
	%	%	%	%	%
A	25.57 ± 8.90 ^{a}	40.93 ± 2.14 ^{a}	20.30 ± 6.68 ^{ab}	1.433 ± 0.81 ^{c}	8.433 ± 1.69 ^{d}
B	5.33 ± 0.60 ^{b}	38.13 ± 1.44 ^{a}	27.37 ± 1.20 ^{a}	4.867 ± 0.29 ^{a}	23.967 ± 0.64 ^{b}
C	0.267 ± 0.06 ^{c}	0.23 ± 0.06 ^{c}	0.20 ± 0.00 ^{d}	0.500 ± 0.00 ^{c}	28.767 ± 1.32 ^{a}
A x B	15.45 ± 4.71 ^{a}	39.53 ± 0.70 ^{a}	23.83 ± 3.88 ^{a}	3.15 ± 0.49 ^{b}	16.200 ± 1.06 ^{c}
A x C	12.92 ± 4.42 ^{a}	20.58 ± 1.09 ^{b}	10.25 ± 3.34 ^{c}	0.967 ± 0.40 ^{c}	18.600 ± 1.10 ^{c}
B x C	2.80 ± 0.28 ^{b}	19.18 ± 0.69 ^{b}	13.78 ± 0.60 ^{bc}	2.683 ± 0.14 ^{b}	26.367 ± 0.46 ^{ab}

Supplementary Table S3. Values of soil chemical properties of donors A, B and C and outcome soils AxB, AxC and BxC. Values are means ± SD; *n* = 3. Means are followed by different superscripted lowercase letters (a-d) that represents soil significant difference from each other at *P* < 0.05 (Tukey's HSD test). OM = organic matter; CEC = cation exchange capacity; TC = total carbon; TN = total nitrogen

Soil	pH	OM	P	K	Mg	Ca
	(H_2O)	%	(ppm)	(ppm)	(ppm)	(ppm)
A	5.80 ± 0.01 ^{b}	0.63 ± 0.02 ^{d}	294.33 ± 32.0 ^{a}	53.33 ± 4.16 ^{c}	71.33 ± 5.50 ^{c}	375.40 ± 36.47 ^{c}
B	5.14 ± 0.03 ^{d}	1.46 ± 0.19 ^{c}	38.33 ± 1.58 ^{c}	29.67 ± 2.08 ^{c}	53.33 ± 2.52 ^{c}	193.97 ± 23.98 ^{c}
C	6.77 ± 0.08 ^{a}	3.64 ± 0.07 ^{a}	122.00 ± 6.08 ^{b}	396.00 ± 16.52 ^{a}	481.33 ± 20.50 ^{a}	3493.63 ± 232.25 ^{a}
A x B	5.44 ± 0.03 ^{c}	1.03 ± 0.07 ^{cd}	109.00 ± 2.00 ^{b}	33.00 ± 1.00 ^{c}	60.50 ± 0.50 ^{c}	202.80 ± 3.20 ^{c}
A x C	5.84 ± 0.10 ^{b}	2.08 ± 0.28 ^{b}	145.00 ± 22.7 ^{b}	220.00 ± 16.82 ^{b}	258.33 ± 24.01 ^{b}	2102.5 ± 123.15 ^{b}
B x C	5.60 ± 0.21 ^{bc}	2.44 ± 0.37 ^{b}	103.33 ± 13.1 ^{b}	217.33 ± 25.32 ^{b}	244.33 ± 37.55 ^{b}	2039.07 ± 345.96 ^{b}

Soil	Zn	Cu	S	NO_3^-	NH_4^+	Soluble Salts
	(ppm)	(ppm)	(ppm)	(ppm)	(mg/kg)	(mmhos/cm)
A	12.33 ± 1.31 ^{c}	6.17 ± 0.60 ^{a}	14.10 ± 1.35 ^{c}	9.100 ± 0.35 ^{c}	4.46 ± 0.12 ^{c}	0.090 ± 0.00 ^{d}
B	3.60 ± 0.17 ^{d}	1.00 ± 0.00 ^{e}	20.67 ± 1.06 ^{b}	1.033 ± 0.06 ^{e}	16.10 ± 0.62 ^{b}	0.077 ± 0.01 ^{d}
C	37.33 ± 2.36 ^{a}	3.57 ± 0.15 ^{bc}	25.93 ± 2.58 ^{a}	3.133 ± 0.23 ^{d}	38.42 ± 1.16 ^{a}	0.313 ± 0.01 ^{b}
A x B	5.95 ± 0.05 ^{d}	2.95 ± 0.05 ^{cd}	13.10 ± 0.00 ^{c}	31.08 ± 1.78 ^{b}	1.06 ± 0.01 ^{d}	0.145 ± 0.01 ^{c}
A x C	20.27 ± 0.81 ^{b}	4.17 ± 0.46 ^{b}	19.07 ± 1.63 ^{b}	160.63 ± 26.79 ^{a}	1.84 ± 0.31 ^{d}	0.640 ± 0.09 ^{a}
B x C	17.10 ± 2.00 ^{b}	2.40 ± 0.30 ^{d}	19.63 ± 0.95 ^{b}	158.08 ± 8.77 ^{a}	2.19 ± 0.45 ^{d}	0.590 ± 0.06 ^{a}

Soil	Acidity index	CEC	TC	TN
	(meq/100g)	(meq/100g)	%	%

A	3.00 ± 0.35 [b]	5.60 ± 0.61 [d]	0.72 ± 0.00 [d]	0.06 ± 0.01 [c]
B	7.70 ± 0.35 [a]	9.20 ± 0.26 [c]	1.29 ± 0.01 [c]	0.08 ± 0.00 [c]
C	1.33 ± 1.15 [c]	21.33 ± 0.99 [a]	2.39 ± 0.05 [a]	0.22 ± 0.02 [a]
A x B	3.9 ± 0.00 [b]	5.5 ± 0.00 [d]	0.92 ± 0.01 [d]	0.06 ± 0.00 [c]
A x C	3.0 ± 0.35 [b]	16.23 ± 0.55 [b]	1.38 ± 0.18 [c]	0.13 ± 0.03 [b]
B x C	4.5 ± 0.60 [b]	17.30 ± 1.83 [b]	1.70 ± 0.21 [b]	0.12 ± 0.00 [b]

Changes in bacterial community alpha diversity following MCC

Changes in bacterial community alpha diversity following MCC are shown in **Supplementary Figure S3** and **Supplementary Table S4**. In brief, observed ASVs and Chao1 index tended to be statistically similar to one or both donors or in between them (Tukey's HSD test, *P* < 0.05). Contradictory results were found in the outcome community BxC^{-1} at day 15 and outcome community BxC^{-3} at days 15 and 30 showing statistically higher values compared to both donors (Tukey's HSD test, *P* < 0.05). Likewise, outcome community bacterial Shannon diversity was often found to be statistically similar to one or both donors but presented statistically higher values at specific time points (Tukey's HSD test, *P* < 0.05). This trend was observed in outcome community AxB^{-1} at day 15, AxB^{-5} at days 1 and 5, BxC^{-1} at day 15, and BxC^{-3} at days 15 and 30. Outcome community evenness, however, often showed higher evenness values compared to either of the donors (Tukey's HSD test, *P* < 0.05). This was observed in outcome communities AxB^{-1} at day 5, 15, and 30, AxB^{-3} throughout all the time points, AxC^{-1} at day 5, and AxC^{-5} at day 30.

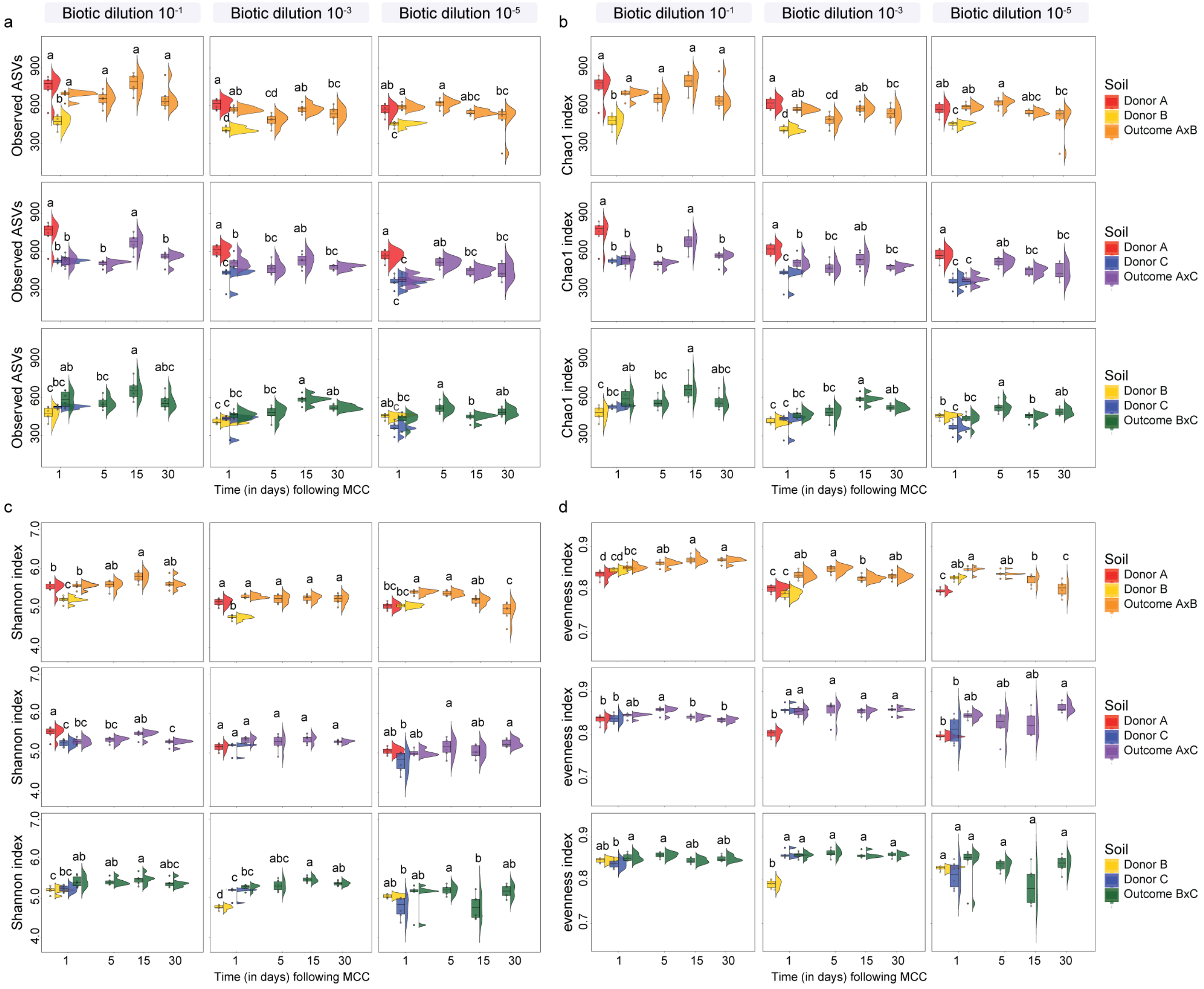

Supplementary Figure S3. Metrics of bacterial community alpha diversity across the biotic dilutions 10^{-1}, 10^{-3} and 10^{-5} treatment sets. (**a**) Bacterial observed ASVs, (**b**) Chao1 index, (**c**) Shannon diversity index, and (**d**) evenness index are colored by soil type and different lowercase letters indicate significant differences at *P* < 0.05 (Tukey's HSD test) between the soils and time

points. Violin plots represent the distribution of the data, and the horizontal bar within the boxplot marks the median.

Supplementary Table S4. Values of the metrics of bacterial community richness following MCC compared to the donors at day 1 (t1). Values are means ± SD; *n* = 6; * *n* = 5; ** *n* = 4. Means are followed by different superscripted lowercase letters (a-d) that represents soil significant difference from each other at $P < 0.05$ (Tukey's HSD test).

**Soils A, B and AxB**	**Observed ASVs**	**Chao1**	**Shannon index**	**Evenness**
**Biotic dilution 10^{-1}**				
A $_{t1}$	742.50 ± 105.33 ^{a}	747.36 ± 107.31 ^{a}	5.51 ± 0.157 ^{b}	0.84 ± 0.010 ^{d}
B $_{t1}$	473.83 ± 51.72 ^{b}	478.61 ± 55.95 ^{b}	5.21 ± 0.087 ^{c}	0.85 ± 0.005 ^{cd}
A x B $_{t1}$	686.50 ± 35.43 ^{a}	692.08 ± 37.58 ^{a}	5.57 ± 0.083 ^{b}	0.85 ± 0.007 ^{bc}
A x B $_{t5}$	654.00 ± 61.21 ^{a}	657.34 ± 61.74 ^{a}	5.58 ± 0.123 ^{ab}	0.86 ± 0.007 ^{ab}
A x B $_{t15}$	778.33 ± 77.17 ^{a}	787.46 ± 78.00 ^{a}	5.79 ± 0.136 ^{a}	0.87 ± 0.008 ^{a}
A x B $_{t30}$	661.17 ± 96.56 ^{a}	668.69 ± 106.88 ^{a}	5.63 ± 0.148 ^{ab}	0.87 ± 0.006 ^{a}
**Biotic dilution 10^{-3}**				
A $_{t1}$	610.50 ± 42.63 ^{a}	617.79 ± 45.38 ^{a}	5.15 ± 0.098 ^{a}	0.80 ± 0.009 ^{c}
B $_{t1}$	412.83 ± 21.94 ^{d}	415.55 ± 24.48 ^{d}	4.76 ± 0.058 ^{b}	0.79 ± 0.010 ^{c}
A x B $_{t1}$	571.50 ± 21.82 ^{ab}	576.21 ± 21.40 ^{ab}	5.29 ± 0.051 ^{a}	0.83 ± 0.010 ^{ab}
A x B $_{t5}$	483.50 ± 50.83 ^{cd}	486.90 ± 54.15 ^{cd}	5.23 ± 0.108 ^{a}	0.85 ± 0.009 ^{a}
A x B $_{t15}$	575.33 ± 35.86 ^{ab}	580.24 ± 37.64 ^{ab}	5.25 ± 0.088 ^{a}	0.83 ± 0.008 ^{b}
A x B $_{t30}$	536.67 ± 56.44 ^{bc}	542.88 ± 57.97 ^{bc}	5.23 ± 0.130 ^{a}	0.83 ± 0.009 ^{ab}
**Biotic dilution 10^{-5}**				
A $_{t1}$	566.00 ± 47.07 ^{ab}	571.09 ± 50.58 ^{ab}	5.05 ± 0.092 ^{bc}	0.80 ± 0.006 ^{c}
B $_{t1}$	453.00 ± 20.94 ^{c}	454.97 ± 21.88 ^{c}	5.06 ± 0.047 ^{bc}	0.83 ± 0.005 ^{ab}
A x B $_{t1}$	592.83 ± 24.29 ^{a}	594.90 ± 24.70 ^{ab}	5.40 ± 0.058 ^{a}	0.85 ± 0.008 ^{a}
A x B $_{t5}$	616.83 ± 35.55 ^{a}	622.63 ± 36.80 ^{a}	5.38 ± 0.065 ^{a}	0.84 ± 0.008 ^{ab}
A x B $_{t15}$	546.67 ± 20.16 ^{abc}	548.20 ± 20.25 ^{abc}	5.19 ± 0.097 ^{ab}	0.82 ± 0.013 ^{b}
A x B $_{t30}$	483.67 ± 131.75 ^{bc}	488.46 ± 134.34 ^{bc}	4.92 ± 0.254 ^{c}	0.80 ± 0.018 ^{c}
**Soils A, C and AxC**				
**Biotic dilution 10^{-1}**				
A $_{t1}$	742.50 ± 105.33 ^{a}	747.36 ± 107.31 ^{a}	5.51 ± 0.157 ^{a}	0.84 ± 0.010 ^{b}
C $_{t1}$	526.17 ± 15.01 ^{b}	529.83 ± 15.33 ^{b}	5.25 ± 0.085 ^{c}	0.84 ± 0.010 ^{b}
A x C $_{t1}$	529.50 ± 49.17 ^{b}	537.47 ± 51.68 ^{b}	5.29 ± 0.085 ^{bc}	0.84 ± 0.006 ^{ab}
A x C $_{t5}$	505.17 ± 25.76 ^{b}	505.89 ± 26.22 ^{b}	5.33 ± 0.074 ^{bc}	0.84 ± 0.005 ^{a}
A x C $_{t15}$	670.67 ± 70.98 ^{a}	678.19 ± 74.40 ^{a}	5.47 ± 0.102 ^{ab}	0.86 ± 0.008 ^{b}
A x C $_{t30}$	552.00 ± 48.56 ^{b}	555.52 ± 49.42 ^{b}	5.26 ± 0.083 ^{c}	0.83 ± 0.005 ^{b}
**Biotic dilution 10^{-3}**				
A $_{t1}$	610.50 ± 42.63 ^{a}	617.79 ± 45.38 ^{a}	5.15 ± 0.098 ^{a}	0.80 ± 0.009 ^{b}
C $_{t1}$	409.50 ± 73.06 ^{c}	411.51 ± 74.02 ^{c}	5.15 ± 0.136 ^{a}	0.86 ± 0.008 ^{a}
A x C $_{t1}$	517.33 ± 50.73 ^{b}	520.89 ± 51.31 ^{b}	5.33 ± 0.085 ^{a}	0.85 ± 0.010 ^{a}
A x C $_{t5}$	472.50 ± 51.80 ^{bc}	475.30 ± 51.78 ^{bc}	5.26 ± 0.206 ^{a}	0.85 ± 0.023 ^{a}
A x C $_{t15}$	529.83 ± 58.73 ^{ab}	535.81 ± 63.03 ^{ab}	5.35 ± 0.110 ^{a}	0.85 ± 0.007 ^{a}
A x C $_{t30}$	478.33 ± 21.08 ^{bc}	480.77 ± 21.69 ^{bc}	5.28 ± 0.042 ^{a}	0.86 ± 0.008 ^{a}
**Biotic dilution 10^{-5}**				
A $_{t1}$	566.00 ± 47.07 ^{a}	571.09 ± 50.58 ^{a}	5.05 ± 0.092 ^{ab}	0.80 ± 0.006 ^{b}
C $_{t1}$	362.33 ± 46.67 ^{c}	363.38 ± 46.94 ^{c}	4.78 ± 0.266 ^{b}	0.81 ± 0.031 ^{b}
A x C $_{t1}$ **	379.50 ± 44.92 ^{c}	380.76 ± 44.72 ^{c}	5.01 ± 0.094 ^{ab}	0.84 ± 0.009 ^{ab}
A x C $_{t5}$	512.83 ± 37.47 ^{ab}	518.72 ± 35.90 ^{ab}	5.14 ± 0.225 ^{a}	0.82 ± 0.033 ^{ab}
A x C $_{t15}$ *	444.60 ± 32.40 ^{bc}	448.54 ± 33.84 ^{bc}	5.04 ± 0.171 ^{ab}	0.83 ± 0.030 ^{ab}
A x C $_{t30}$ *	444.00 ± 71.31 ^{bc}	445.14 ± 71.77 ^{bc}	5.25 ± 0.099 ^{a}	0.86 ± 0.012 ^{a}
**Soils B, C and BxC**				
**Biotic dilution 10^{-1}**				

B $_{t1}$	473.83 ± 51.72 ^{c}	478.61 ± 55.95 ^{c}	5.21 ± 0.087 ^{c}	0.85 ± 0.005 ^{ab}
C $_{t1}$	526.17 ± 15.01 ^{bc}	529.83 ± 15.33 ^{bc}	5.25 ± 0.085 ^{bc}	0.84 ± 0.010 ^{b}
B x C $_{t1}$	588.00 ± 65.04 ^{ab}	591.13 ± 66.36 ^{ab}	5.43 ± 0.145 ^{ab}	0.85 ± 0.009 ^{a}
B x C $_{t5}$	559.00 ± 48.62 ^{bc}	562.00 ± 49.17 ^{bc}	5.42 ± 0.086 ^{ab}	0.86 ± 0.007 ^{a}
B x C $_{t15}$	661.50 ± 80.66 ^{a}	671.03 ± 89.50 ^{a}	5.48 ± 0.133 ^{a}	0.84 ± 0.007 ^{ab}
B x C $_{t30}$	568.67 ± 62.59 ^{abc}	571.74 ± 62.25 ^{abc}	5.39 ± 0.104 ^{abc}	0.85 ± 0.007 ^{ab}
**Biotic dilution 10^{-3}**				
B $_{t1}$	412.83 ± 21.94 ^{c}	415.55 ± 24.48 ^{c}	4.76 ± 0.058 ^{d}	0.79 ± 0.010 ^{b}
C $_{t1}$	409.50 ±73.06 ^{c}	411.51 ± 74.02 ^{c}	5.15 ± 0.136 ^{c}	0.86 ± 0.008 ^{a}
B x C $_{t1}$	465.17 ± 30.45 ^{bc}	467.97 ± 31.33 ^{bc}	5.27 ± 0.073 ^{bc}	0.86 ± 0.007 ^{a}
B x C $_{t5}$	476.67 ± 56.28 ^{bc}	479.57 ± 57.22 ^{bc}	5.31 ± 0.139 ^{abc}	0.86 ± 0.010 ^{a}
B x C $_{t15}$	585.33 ± 38.68 ^{a}	591.29 ± 39.49 ^{a}	5.46 ± 0.073 ^{a}	0.86 ± 0.007 ^{a}
B x C $_{t30}$	521.83 ± 21.93 ^{ab}	524.69 ± 23.55 ^{ab}	5.37 ± 0.056 ^{ab}	0.86 ± 0.005 ^{a}
**Biotic dilution 10^{-5}**				
B $_{t1}$	453.00 ± 20.94 ^{ab}	454.97 ± 21.88 ^{b}	5.06 ± 0.047 ^{ab}	0.83 ± 0.005 ^{a}
C $_{t1}$	362.33 ± 46.67 ^{c}	363.38 ± 46.94 ^{c}	4.78 ± 0.266 ^{b}	0.81 ± 0.031 ^{a}
B x C $_{t1}$	427.50 ± 54.49 ^{bc}	429.81 ± 54.93 ^{bc}	5.07 ± 0.371 ^{ab}	0.84 ± 0.045 ^{a}
B x C $_{t5}$	520.67 ± 41.67 ^{a}	527.08 ± 46.17 ^{a}	5.21 ± 0.122 ^{a}	0.83 ± 0.013 ^{a}
B x C $_{t15}$	444.83 ± 33.70 ^{b}	449.86 ± 33.50 ^{b}	4.79 ± 0.315 ^{b}	0.79 ± 0.045 ^{a}
B x C $_{t30}$	486.50 ± 32.75 ^{ab}	490.60 ± 31.43 ^{ab}	5.18 ± 0.164 ^{ab}	0.84 ± 0.019 ^{a}

Changes in community taxa membership following MCC

Variations in community taxa membership were observed from day 1 to day 30 following MCC (**Supplementary Figure S4**). Venn diagrams were used to depict that shared ASVs among the time points ranged between 26 to 36% of total ASVs within the outcome community and biotic dilutions. Across the 30 days sampling period, AxB^{-1} shared 26% (709 ASVs); AxC^{-1}, 28% (614 ASVs); BxC^{-1}, 32% (650 ASVs); AxB^{-3} shared 28% (543 ASVs); AxC^{-3}, 28% (571 ASVs); BxC^{-3}, 30% (559 ASVs); AxB^{-5}, 36% (614 ASVs), AxC^{-5}, 26% (406 ASVs); and BxC^{-5}, 30% (508 ASVs). Additionally, unique ASVs accounted for 6 to 16% of total bacterial sequences, indicating the appearance and disappearance of distinct taxa over time. Specifically, the number of unique taxa at days 1, 5, 15, and 30 was 276, 215, 306, and 285 for AxB^{-1}, 213, 155, 357 and 234 for AxC^{-1}, and 229, 192, 246, 169 for BxC^{-1}, respectively. In the intermediate biotic dilution (i.e., 10^{-3}), AxB^{-3} had 276, 114, 195, and 193 unique taxa, AxC^{-3} presented 290, 222, 218, and 181, and BxC^{-3} showed 184, 171, 231, and 205 at days 1, 5, 15, and 30 respectively. Additionally, AxB^{-5} presented 181, 189, 128, and 151 unique ASVs at days 1, 5, 15, and 30 respectively, while AxC^{-5} showed 107, 229, 165 and 170, and BxC^{-5} 154, 181, 153, and 189.

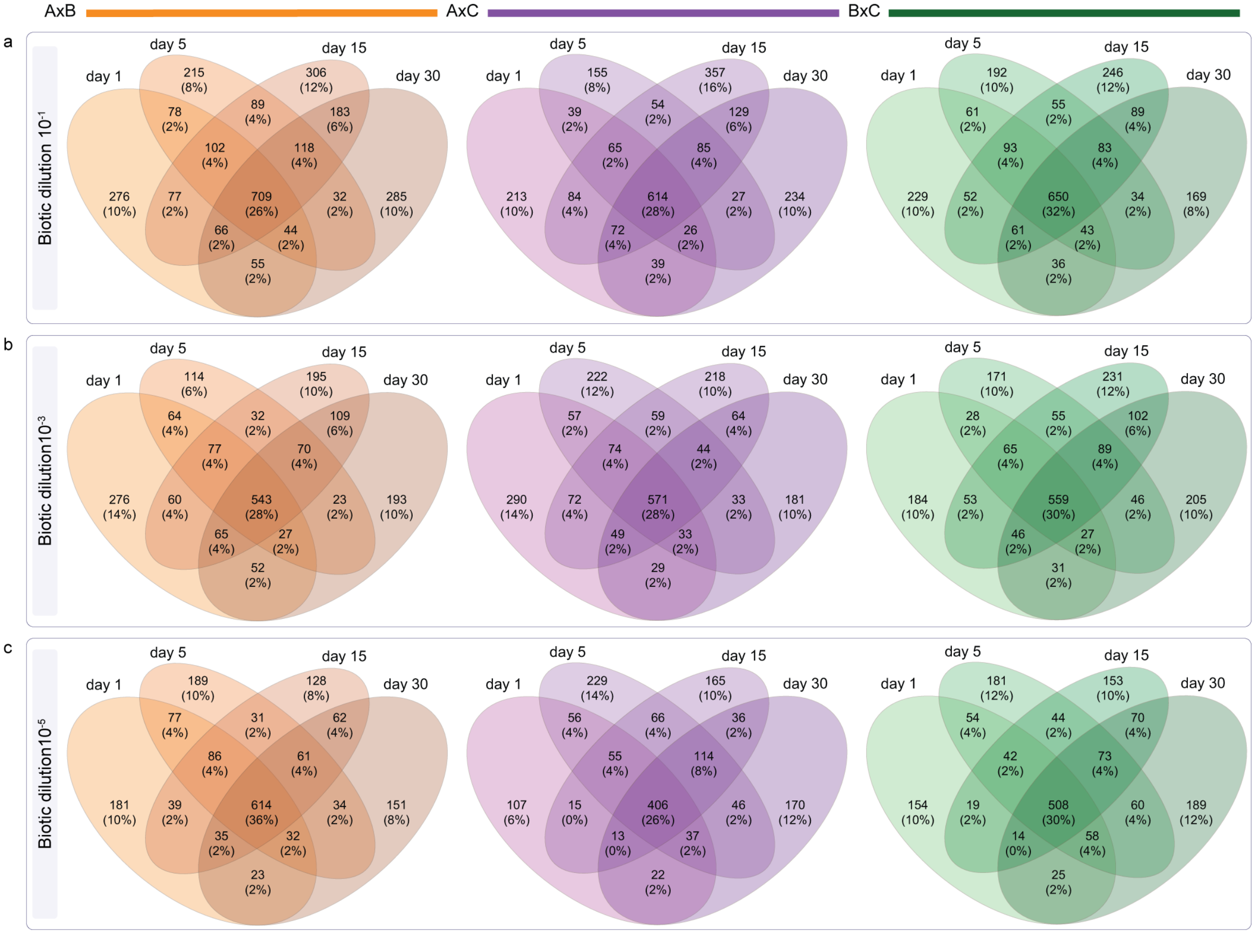

Supplementary Figure S4. Venn Diagram displaying the number of unique and shared taxa present in the outcome community over time following MCC for (**a**) biotic dilution 10^{-1}, (**b**) biotic dilution 10^{-3}, and (**c**) biotic dilution 10^{-5} treatment sets. Outcome community AxB is shown in orange, AxC in purple, and BxC in green. The number of taxa is followed by the corresponding percentage of the total taxa present within the treatment across all the time points.

Quantification of donor contributions to outcome soil properties following MCC

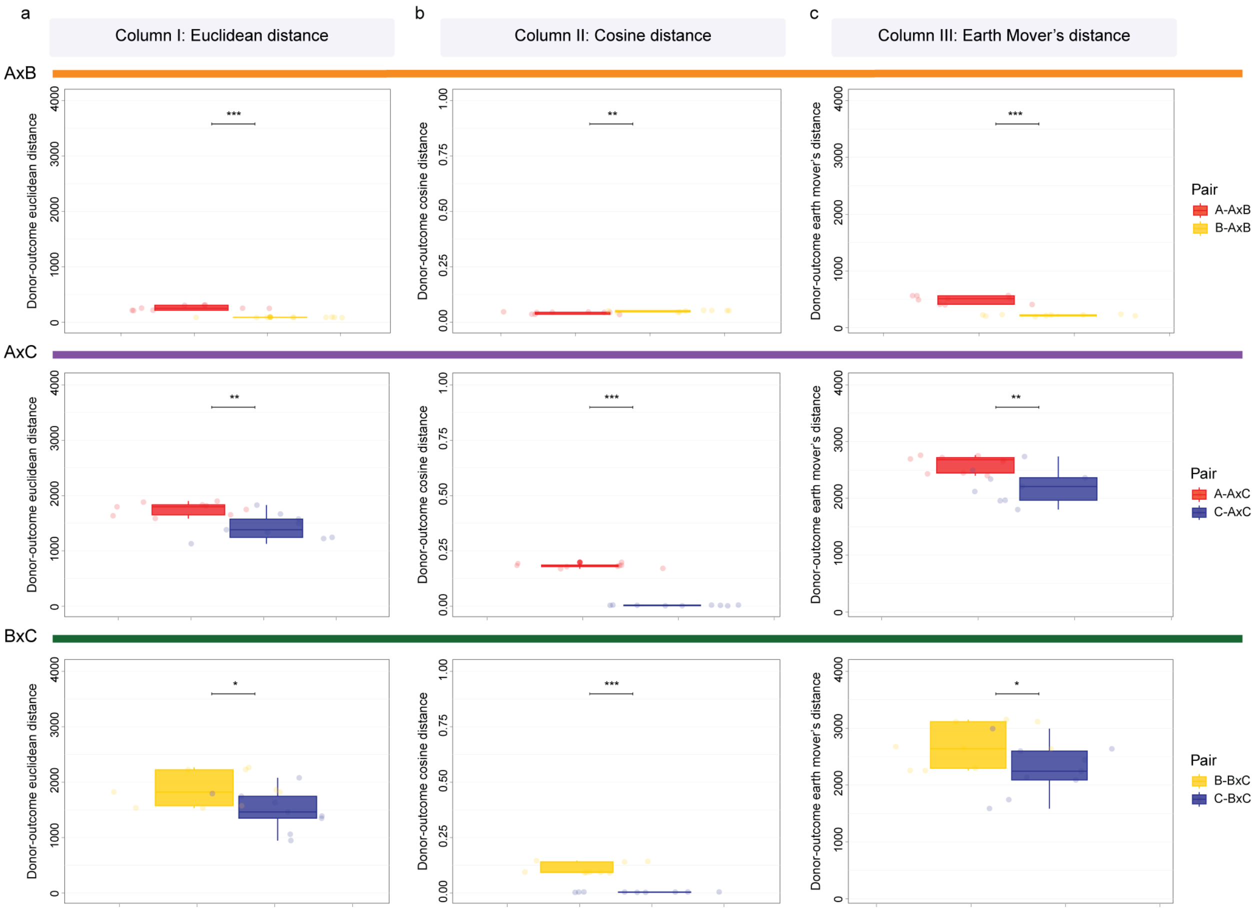

Supplementary Figure S5. Estimates of the contribution of donor soil properties to the outcome soil based on (**a**) Euclidean, (**b**) Cosine, and (**c**) Earth Mover's distances. Statistical differences in the relative contribution of donors to outcome were assessed by non-parametric Mann Whitney U and are denoted by an asterisk above the boxplot ("ns", *P* > 0.05; "*", *P* < 0.05; "**", *P* < 0.01; "***", *P* < 0.001). Lower distances represent a greater contribution of the donor to the outcome community (i.e., dominance).

Supplementary Table S5. Contribution of donors to outcome soil properties based on Jensen-Shannon divergence, Euclidean, Cosine, and Earth Mover's distances. Values are means ± SD; *n* = 9. Statistical differences were determined using non-parametric Mann Whitney U test at α = 0.05.

**Outcome Soil**	**Distances**	**Donor – Outcome**		**Mann Whitney U test**
**AxB**		**A – AxB**	**B – AxB**	
	Jensen-Shannon Divergence	0.169 ± 0.012	0.203 ± 0.005	P = 0.0004122948
	Euclidean	257.25 ± 41.40	86.40 ± 2.98	P = 0.0004122948
	Cosine	0.042 ± 0.005	0.050 ± 0.004	P = 0.003568628
	Earth Mover's	494.41 ± 68.52	221.05 ± 13.83	P = 0.0004122948
**AxC**		**A – AxC**	**C – AxC**	
	Jensen-Shannon Divergence	0.335 ± 0.009	0.168 ± 0.013	P = 0.0004122948
	Euclidean	1760.20 ± 112.26	1431.38 ± 228.47	P = 0.006193318
	Cosine	0.183 ± 0.009	0.004 ± 0.002	P = 0.0004122948
	Earth Mover's	2613.70 ± 145.04	2220.94 ± 294.34	P = 0.008071487
**BxC**		**B – BxC**	**C – BxC**	
	Jensen-Shannon Divergence	0.362 ± 0.015	0.163 ± 0.011	P = 0.0004122948
	Euclidean	1874.00 ± 301.67	1495.34 ± 359.90	P = 0.02727538
	Cosine	0.109 ± 0.025	0.004 ± 0.001	P = 0.0004122948
	Earth Mover's	2682.40 ± 373.26	2273.99 ± 446.17	P = 0.02727538

Quantification of donor contributions to outcome community following MCC

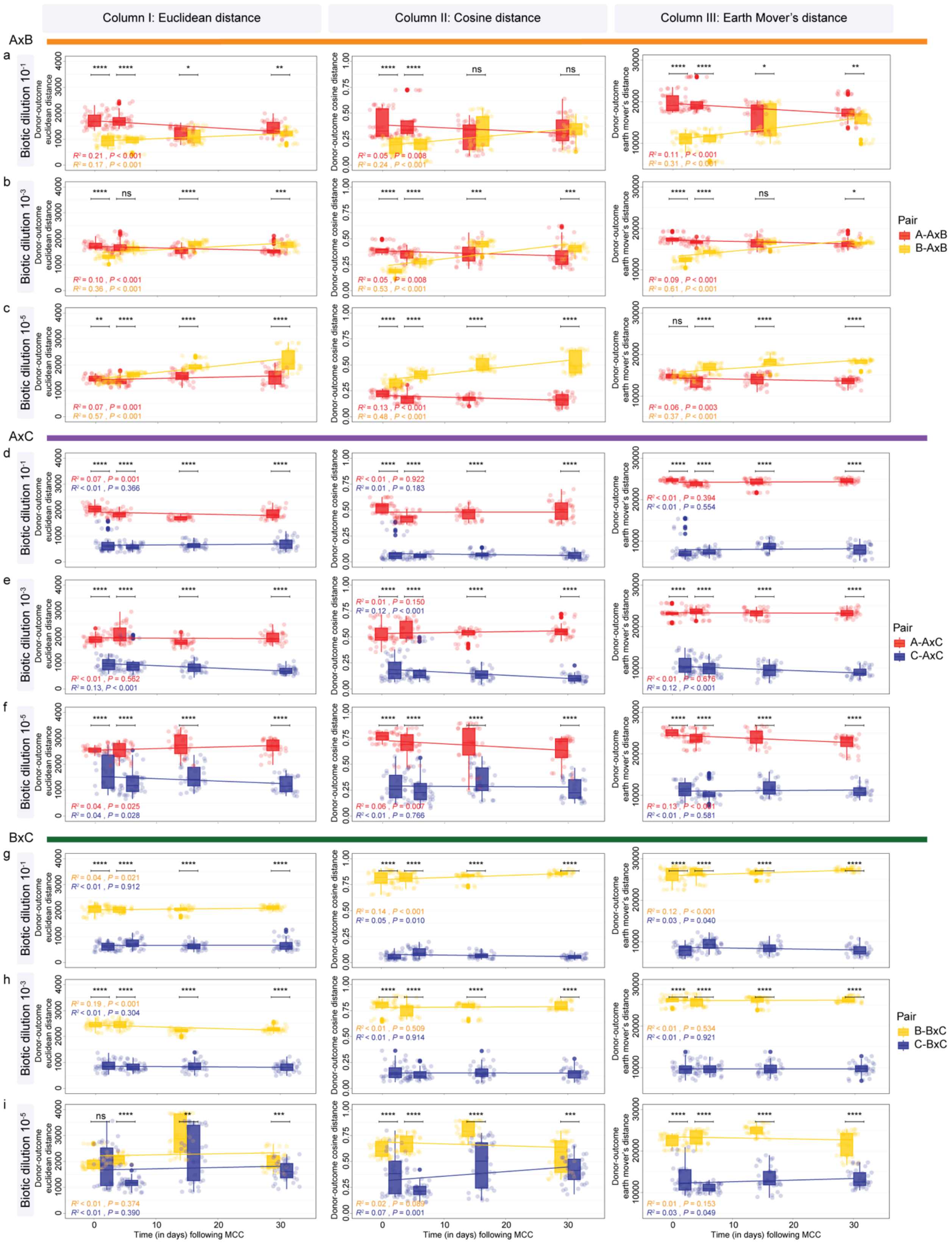

Supplementary Figure S6. Boxplots and linear regressions displaying the relative contribution of donors to the outcome community structure based on Euclidean (Column I), Cosine (Column II), and Earth Mover's distances (Column III) over time based on donor-outcome distances (**a**) AxB at the biotic dilution 10^{-1}, (**b**) AxB at the biotic dilution 10^{-3}, (**c**) AxB at the biotic dilution 10^{-5}, (**d**) AxC at the biotic dilution 10^{-1}, (**e**) AxC at the biotic dilution 10^{-3}, (**f**) AxC at the biotic dilution 10^{-5}, (**g**) BxC at the biotic dilution 10^{-1}, (**h**) BxC at the biotic dilution 10^{-3}, and (**i**) BxC at the biotic dilution 10^{-5}. Statistical differences in the relative contributions of donors to outcome were assessed by non-parametric Mann Whitney U and are denoted by an asterisk above the boxplot ("ns", $P > 0.05$; "*", $P < 0.05$; "**", $P < 0.01$; "***", $P < 0.001$; "****", $P < 0.0001$). Lower distances represent a greater contribution of the donor to the outcome community (i.e., dominance).

Supplementary Table S6. Contribution of donors to outcome communities based on Jensen-Shannon divergence, Euclidean, Cosine, and Earth Mover's distances over time. Values are means ± SD; *n* = 288. Statistical differences were determined using non-parametric Mann Whitney U test at $\alpha = 0.05$.

**Outcome Community**	**Time (days)**	**Distances**	**Donor – Outcome**		**Mann Whitney U test**
**AxB^{-1}**			**A^{-1} – AxB^{-1}**	**B^{-1} – AxB^{-1}**	
	1	Jensen-Shannon Divergence	0.595 ± 0.040	0.398 ± 0.037	*P* = 3.05 e-13
	1	Euclidean	1721.98 ± 273.89	964.10 ± 199.72	*P* = 3.05 e-13
	1	Cosine	0.386 ± 0.131	0.195 ± 0.073	*P* = 1.16 e-11
	1	Earth Mover's	19648.06 ± 2091.06	11018.61 ± 1639.39	*P* = 3.05 e-13
	5	Jensen-Shannon Divergence	0.597 ± 0.060	0.393 ± 0.067	*P* = 3.05 e-13
	5	Euclidean	1745.10 ± 286.36	911.48 ± 235.72	*P* = 3.05 e-13
	5	Cosine	0.403 ± 0.152	0.187 ± 0.077	*P* = 3.05 e-13
	5	Earth Mover's	19885.50 ± 2853.85	10749.06 ± 2550.44	*P* = 3.05 e-13
	15	Jensen-Shannon Divergence	0.537 ± 0.064	0.479 ± 0.080	*P* = 0.001523
	15	Euclidean	1254.33 ± 259.36	1100.49 ± 270.57	*P* = 0.040943
	15	Cosine	0.290 ± 0.124	0.307 ± 0.147	*P* = 0.664580
	15	Earth Mover's	16682.56 ± 3124.48	14572.56 ± 3888.03	*P* = 0.043220
	30	Jensen-Shannon Divergence	0.561 ± 0.047	0.499 ± 0.060	*P* = 0.000017
	30	Euclidean	1388.21 ± 261.87	1196.60 ± 212.12	*P* = 0.005501
	30	Cosine	0.326 ± 0.126	0.332 ± 0.102	*P* = 0.427200
	30	Earth Mover's	17635.22 ± 2300.04	15415.17 ± 2565.48	*P* = 0.001252
**AxB^{-3}**			**A^{-3} – AxB^{-3}**	**B^{-3} – AxB^{-3}**	
	1	Jensen-Shannon Divergence	0.544 ± 0.017	0.428 ± 0.019	*P* = 3.05 e-13
	1	Euclidean	1730.26 ± 128.10	1300.21 ± 133.01	*P* = 3.60 e-13
	1	Cosine	0.401 ± 0.047	0.181 ± 0.033	*P* = 3.05 e-13
	1	Earth Mover's	17504.72 ± 911.66	12264.56 ± 858.81	*P* = 3.05 e-13
	5	Jensen-Shannon Divergence	0.528 ± 0.011	0.481 ± 0.012	*P* = 3.31 e-13
	5	Euclidean	1675.38 ± 232.51	1619.98 ± 120.92	*P* = 8.35 e-01
	5	Cosine	0.349 ± 0.053	0.286 ± 0.040	*P* = 3.58 e-06
	5	Earth Mover's	16709.89 ± 595.11	14357.94 ± 631.52	*P* = 4.62 e-13
	15	Jensen-Shannon Divergence	0.522 ± 0.025	0.530 ± 0.021	*P* = 0.063933

	15	Euclidean	1552.75 ± 154.32	1783.29 ± 140.57	P = 8.55 e-08
	15	Cosine	0.362 ± 0.093	0.437 ± 0.053	P = 0.000246
	15	Earth Mover's	16537.00 ± 1275.04	16431.83 ± 900.42	P = 0.923735
	30	Jensen-Shannon Divergence	0.522 ± 0.026	0.530 ± 0.012	P = 0.000840
	30	Euclidean	1573.01 ± 194.27	1736.55 ± 138.96	P = 0.000105
	30	Cosine	0.335 ± 0.113	0.401 ± 0.048	P = 0.000120
	30	Earth Mover's	16394.39 ± 1305.13	16479.33 ± 425.89	P = 0.018863
**AxB^{-5}**			**A^{-5} – AxB^{-5}**	**B^{-5} – AxB^{-5}**	
	1	Jensen-Shannon Divergence	0.479 ± 0.016	0.481 ± 0.017	P = 0.308090
	1	Euclidean	1474.12 ± 116.85	1376.56 ± 120.82	P = 0.001645
	1	Cosine	0.221 ± 0.030	0.316 ± 0.041	P = 2.57 e-12
	1	Earth Mover's	14864.50 ± 694.96	14749.44 ± 827.60	P = 0.995507
	5	Jensen-Shannon Divergence	0.447 ± 0.026	0.529 ± 0.019	P = 3.05 e-13
	5	Euclidean	1363.71 ± 166.28	1619.19 ± 98.34	P = 9.57 e-09
	5	Cosine	0.182 ± 0.053	0.407 ± 0.040	P = 3.05 e-13
	5	Earth Mover's	13429.78 ± 1219.26	16800.78 ± 1019.06	P = 5.92 e-13
	15	Jensen-Shannon Divergence	0.461 ± 0.030	0.560 ± 0.033	P = 1.35 e-12
	15	Euclidean	1559.15 ± 194.29	1959.97 ± 171.57	P = 1.07 e-11
	15	Cosine	0.172 ± 0.035	0.499 ± 0.059	P = 3.05 e-13
	15	Earth Mover's	14133.56 ± 1380.19	18043.78 ± 1615.92	P = 4.28 e-11
	30	Jensen-Shannon Divergence	0.458 ± 0.029	0.577 ± 0.008	P = 3.05 e-13
	30	Euclidean	1563.05 ± 301.81	2160.58 ± 420.97	P = 5.51 e-08
	30	Cosine	0.164 ± 0.058	0.517 ± 0.099	P = 3.05 e-13
	30	Earth Mover's	13545.28 ± 907.57	18036.06 ± 1019.89	P = 3.05 e-13
**AxC^{-1}**			**A^{-1} – AxC^{-1}**	**C^{-1} – AxC^{-1}**	
	1	Jensen-Shannon Divergence	0.707 ± 0.009	0.317 ± 0.062	P = 3.05 e-13
	1	Euclidean	2047.54 ± 152.65	704.34 ± 356.16	P = 3.05 e-13
	1	Cosine	0.534 ± 0.054	0.098 ± 0.112	P = 3.05 e-13
	1	Earth Mover's	24675.00 ± 400.72	8008.33 ± 2902.19	P = 3.05 e-13
	5	Jensen-Shannon Divergence	0.691 ± 0.014	0.311 ± 0.016	P = 3.05 e-13
	5	Euclidean	1840.88 ± 133.77	582.00 ± 118.78	P = 3.05 e-13
	5	Cosine	0.425 ± 0.044	0.057 ± 0.023	P = 3.05 e-13
	5	Earth Mover's	23809.06 ± 534.05	7420.28 ± 781.70	P = 3.05 e-13
	15	Jensen-Shannon Divergence	0.696 ± 0.03	0.345 ± 0.026	P = 3.05 e-13
	15	Euclidean	1691.73 ± 76.49	648.99 ± 138.36	P = 3.05 e-13
	15	Cosine	0.467 ± 0.054	0.074 ± 0.031	P = 3.05 e-13
	15	Earth Mover's	23936.28 ± 1150.58	8835.56 ± 1151.69	P = 3.05 e-13
	30	Jensen-Shannon Divergence	0.717 ± 0.015	0.317 ± 0.031	P = 3.05 e-13
	30	Euclidean	1868.33 ± 193.69	702.04 ± 221.14	P = 3.05 e-13
	30	Cosine	0.496 ± 0.094	0.064 ± 0.035	P = 3.05 e-13
	30	Earth Mover's	24530.83 ± 548.50	7870.22 ± 1458.12	P = 3.05 e-13
**AxC^{-3}**			**A^{-3} – AxC^{-3}**	**C^{-3} – AxC^{-3}**	
	1	Jensen-Shannon Divergence	0.668 ± 0.032	0.382 ± 0.052	P = 3.05 e-13

	1	Euclidean	1908.75 ± 165.27	929.76 ± 243.61	*P* = 3.05 e-13
	1	Cosine	0.522 ± 0.103	0.183 ± 0.103	*P* = 4.25 e-13
	1	Earth Mover's	23184.28 ± 1390.23	10651.06 ± 2222.92	*P* = 3.05 e-13
	5	Jensen-Shannon Divergence	0.675± 0.027	0.373 ± 0.042	*P* = 3.05 e-13
	5	Euclidean	2103.84 ± 338.97	1013.50 ± 471.38	*P* = 1.23 e-09
	5	Cosine	0.562 ± 0.089	0.185 ± 0.135	*P* = 8.46 e-12
	5	Earth Mover's	23510.11 ± 1099.41	10058.06 ± 1649.08	*P* = 3.05 e-13
	15	Jensen-Shannon Divergence	0.676 ± 0.025	0.351 ± 0.036	*P* = 3.05 e-13
	15	Euclidean	1811.51 ± 163.17	817.59 ± 187.73	*P* = 3.05 e-13
	15	Cosine	0.528 ± 0.056	0.136 ± 0.052	*P* = 3.05 e-13
	15	Earth Mover's	23222.11 ± 1065.14	9530.50 ± 1563.08	*P* = 3.05 e-13
	30	Jensen-Shannon Divergence	0.675 ± 0.021	0.342 ± 0.026	*P* = 3.05 e-13
	30	Euclidean	1977.34 ± 210.75	698.10 ± 132.67	*P* = 3.05 e-13
	30	Cosine	0.565 ± 0.073	0.104 ± 0.037	*P* = 3.05 e-13
	30	Earth Mover's	23223.33 ± 916.32	8944.39 ± 1117.88	*P* = 3.05 e-13
**AxC^{-5}**			**A^{-5} – AxC^{-5}**	**C^{-5} – AxC^{-5}**	
	1	Jensen-Shannon Divergence	0.706 ± 0.024	0.379 ± 0.050	*P* = 3.06 e-09
	1	Euclidean	2549.10 ± 114.87	1623.59 ± 647.97	*P* = 1.08 e-06
	1	Cosine	0.763 ± 0.049	0.292 ± 0.132	*P* = 3.06 e-09
	1	Earth Mover's	25124.92 ± 923.42	11268.50 ± 2057.00	*P* = 3.06 e-09
	5	Jensen-Shannon Divergence	0.664 ± 0.041	0.374 ± 0.048	*P* = 3.05 e-13
	5	Euclidean	2508.25 ± 310.57	1336.92 ± 498.49	*P* = 2.32 e-11
	5	Cosine	0.686 ± 0.120	0.259 ± 0.128	*P* = 1.14 e-12
	5	Earth Mover's	23554.44 ± 1705.78	10538.89 ± 2111.32	*P* = 3.05 e-13
	15	Jensen-Shannon Divergence	0.678 ± 0.040	0.396 ± 0.040	*P* = 3.02 e-11
	15	Euclidean	2757.60 ± 441.02	1537.41 ± 461.60	*P* = 4.20 e-10
	15	Cosine	0.673 ± 0.206	0.338 ± 0.137	*P* = 9.83 e-08
	15	Earth Mover's	23904.33± 1939.08	11721.83 ± 1963.63	*P* = 3.02 e-11
	30	Jensen-Shannon Divergence	0.654 ± 0.041	0.397 ± 0.040	*P* = 3.02 e-11
	30	Euclidean	2674.40 ± 284.40	1214.04 ± 307.31	*P* = 3.02 e-11
	30	Cosine	0.639 ± 0.136	0.259 ± 0.109	*P* = 1.96 e-10
	30	Earth Mover's	22639.30 ± 1949.53	11060.30 ± 1463.41	*P* = 3.02 e-11
**BxC^{-1}**			**B^{-1} – BxC^{-1}**	**C^{-1} – BxC^{-1}**	
	1	Jensen-Shannon Divergence	0.724 ± 0.051	0.332 ± 0.032	*P* = 3.05 e-13
	1	Euclidean	2056.40 ± 181.54	606.68 ± 154.18	*P* = 3.05 e-13
	1	Cosine	0.802 ± 0.082	0.058 ± 0.030	*P* = 3.05 e-13
	1	Earth Mover's	25989.39 ± 2007.05	7938.39 ± 1239.47	*P* = 3.05 e-13
	5	Jensen-Shannon Divergence	0.731 ± 0.034	0.362 ± 0.029	*P* = 3.05 e-13
	5	Euclidean	2035.08 ± 96.61	739.09 ± 180.45	*P* = 3.05 e-13
	5	Cosine	0.810 ± 0.052	0.097 ± 0.046	*P* = 3.05 e-13
	5	Earth Mover's	26278.33 ± 1404.40	9360.94 ± 1386.11	*P* = 3.05 e-13
	15	Jensen-Shannon Divergence	0.733 ± 0.022	0.340 ± 0.029	*P* = 3.05 e-13
	15	Euclidean	2026.23 ± 133.73	622.00 ± 131.26	*P* = 3.05 e-13

	15	Cosine	0.823 ± 0.042	0.068 ± 0.029	*P* = 3.05 e-13
	15	Earth Mover's	26397.78 ± 879.26	8413.83 ± 1157.09	*P* = 3.05 e-13
	30	Jensen-Shannon Divergence	0.759 ± 0.013	0.323 ± 0.027	*P* = 3.05 e-13
	30	Euclidean	2116.22 ± 77.76	668.41 ± 209.23	*P* = 3.05 e-13
	30	Cosine	0.860 ± 0.019	0.054 ± 0.019	*P* = 3.05 e-13
	30	Earth Mover's	27339.00 ± 445.11	7901.94 ± 1259.08	*P* = 3.05 e-13
**BxC^{-3}**			**B^{-3} – BxC^{-3}**	**C^{-3} – BxC^{-3}**	
	1	Jensen-Shannon Divergence	0.730 ± 0.027	0.364 ± 0.042	*P* = 3.05 e-13
	1	Euclidean	2444.35 ± 147.09	868.55 ± 202.95	*P* = 3.05 e-13
	1	Cosine	0.791 ± 0.066	0.158 ± 0.077	*P* = 3.05 e-13
	1	Earth Mover's	26205.39 ± 1012.31	9734.33 ± 1655.22	*P* = 3.04 e-13
	5	Jensen-Shannon Divergence	0.728 ± 0.031	0.368 ± 0.030	*P* = 3.05 e-13
	5	Euclidean	2455.31 ± 161.02	830.71 ± 163.30	*P* = 3.05 e-13
	5	Cosine	0.770 ± 0.071	0.136 ± 0.052	*P* = 3.05 e-13
	5	Earth Mover's	26086.56 ± 1120.97	9721.11 ± 1251.73	*P* = 3.05 e-13
	15	Jensen-Shannon Divergence	0.731 ± 0.027	0.359 ± 0.035	*P* = 3.05 e-13
	15	Euclidean	2216.33 ± 99.73	842.96 ± 199.86	*P* = 3.05 e-13
	15	Cosine	0.781 ± 0.059	0.155 ± 0.067	*P* = 3.05 e-13
	15	Earth Mover's	26217.56 ± 1128.04	9676.06 ± 1499.75	*P* = 3.05 e-13
	30	Jensen-Shannon Divergence	0.731 ± 0.018	0.362 ± 0.034	*P* = 3.05 e-13
	30	Euclidean	2293.28 ± 113.04	813.82 ± 169.94	*P* = 3.05 e-13
	30	Cosine	0.791 ± 0.041	0.147 ± 0.054	*P* = 3.05 e-13
	30	Earth Mover's	26285.00 ± 694.07	9704.78 ± 1393.96	*P* = 3.05 e-13
**BxC^{-5}**			**B^{-5} – BxC^{-5}**	**C^{-5} – BxC^{-5}**	
	1	Jensen-Shannon Divergence	0.651 ± 0.030	0.440 ± 0.072	*P* = 3.05 e-13
	1	Euclidean	1991.06 ± 338.57	1849.02 ± 780.61	*P* = 0.569528
	1	Cosine	0.616 ± 0.078	0.368 ± 0.190	*P* = 7.09 e-08
	1	Earth Mover's	22623.50 ± 1452.89	13136.50 ± 3447.02	*P* = 1.35 e-12
	5	Jensen-Shannon Divergence	0.659 ± 0.041	0.393 ± 0.029	*P* = 3.05 e-13
	5	Euclidean	2074.64 ± 187.28	1167.57 ± 226.50	*P* = 4.25 e-13
	5	Cosine	0.674 ± 0.084	0.219 ± 0.066	*P* = 3.05 e-13
	5	Earth Mover's	23026.50 ± 1922.81	11076.33 ± 970.28	*P* = 3.05 e-13
	15	Jensen-Shannon Divergence	0.707 ± 0.031	0.438 ± 0.056	*P* = 3.05 e-13
	15	Euclidean	2982.84 ± 690.50	2271.76 ± 1000.89	*P* = 0.001464
	15	Cosine	0.797 ± 0.082	0.475 ± 0.234	*P* = 1.07 e-09
	15	Earth Mover's	24944.61 ± 1286.24	13333.39 ± 2728.49	*P* = 3.05 e-13
	30	Jensen-Shannon Divergence	0.643 ± 0.059	0.446 ± 0.048	*P* = 3.05 e-13
	30	Euclidean	2014.80 ± 360.78	1631.99 ± 364.18	*P* = 0.000144
	30	Cosine	0.568 ± 0.169	0.417 ± 0.126	*P* = 0.003645
	30	Earth Mover's	21780.72 ± 3031.58	13306.06 ± 2102.19	*P* = 1.14 e-12

Quantification of donor contributions to outcome carbon metabolic profiles following MCC

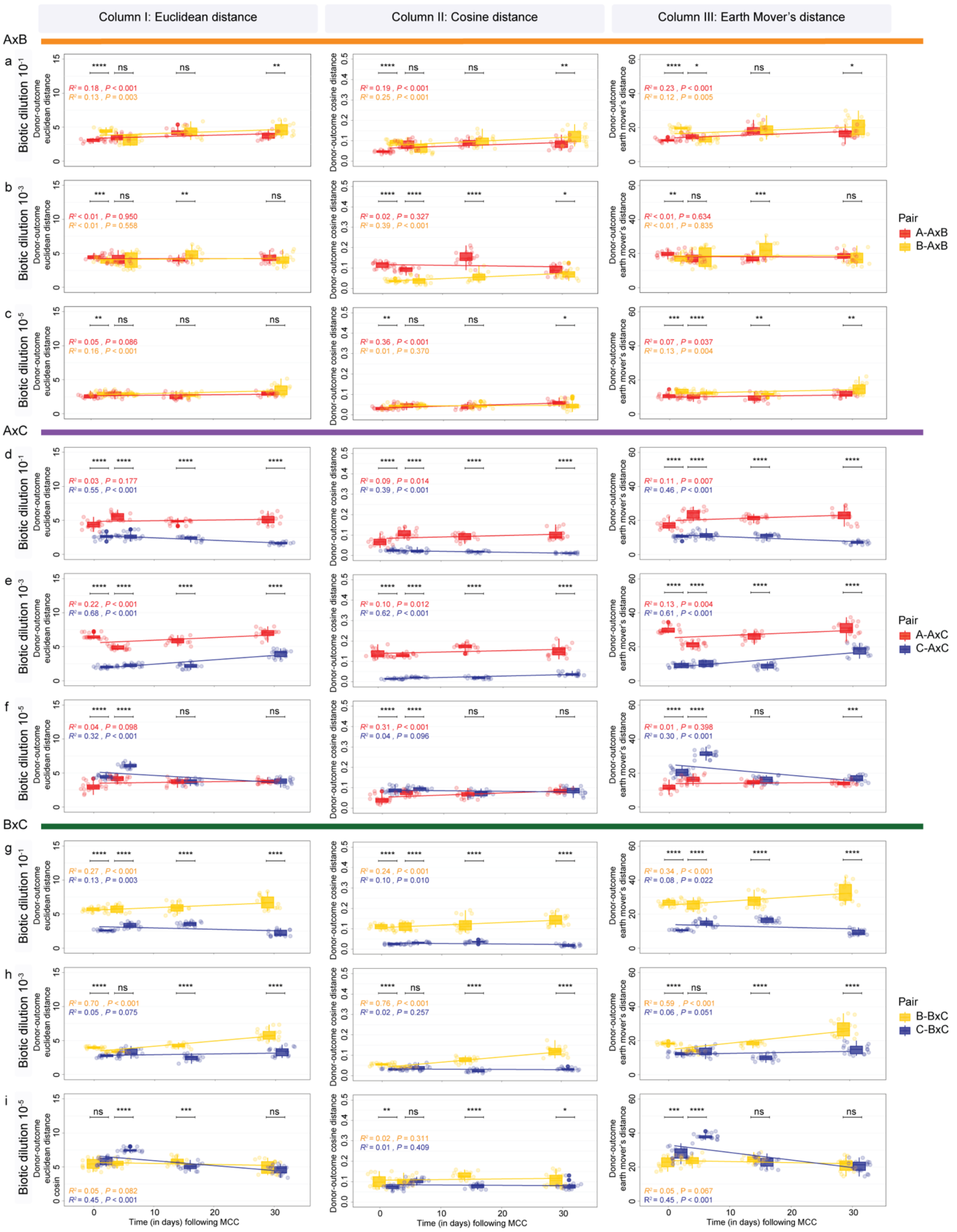

Supplementary Figure S7. Boxplots and linear regressions displaying the relative contribution of donors to the outcome carbon metabolic profiles based on Euclidean (Column I), Cosine (Column II), and Earth Mover's distances (Column III) over time based on donor-outcome distances (**a**) AxB at the biotic dilution 10^{-1}, (**b**) AxB at the biotic dilution 10^{-3}, (**c**) AxB at the biotic dilution 10^{-5}, (**d**) AxC at the biotic dilution 10^{-1}, (**e**) AxC at the biotic dilution 10^{-3}, (**f**) AxC at the biotic dilution 10^{-5}, (**g**) BxC at the biotic dilution 10^{-1}, (**h**) BxC at the biotic dilution 10^{-3}, and (**i**) BxC at biotic dilution 10^{-5}. Statistical differences in the relative contributions of donors to outcome were assessed by non-parametric Mann Whitney U and are denoted by an asterisk above the boxplot ("ns", $P > 0.05$; "*", $P < 0.05$; "**", $P < 0.01$; "***", $P < 0.001$; "****", $P < 0.0001$). Lower distances represent a greater contribution of the donor to the outcome community (i.e., dominance).

Supplementary Table S7. Contributions of donors to outcome carbon metabolism based on Jensen-Shannon divergence, Euclidean, Cosine, and Earth Mover's distances over time. Values are means ± SD; n = 128. Statistical differences were determined using non-parametric Mann Whitney U test at $\alpha = 0.05$.

Outcome Carbon metabolism	Time (days)	Distances	Donor – Outcome		Mann Whitney U test
**AxB^{-1}**			**A^{-1} – AxB^{-1}**	**B^{-1} – AxB^{-1}**	
	1	Jensen-Shannon Divergence	0.162 ± 0.014	0.202 ± 0.010	P = 1.55 e-06
	1	Euclidean	3.084 ± 0.240	4.43 ± 0.331	P = 1.55 e-06
	1	Cosine	0.047 ± 0.007	0.093 ± 0.011	P = 1.55 e-06
	1	Earth Mover's	12.55 ± 0.953	19.55 ± 1.285	P = 1.55 e-06
	5	Jensen-Shannon Divergence	0.215 ± 0.028	0.169 ± 0.018	P = 0.000096
	5	Euclidean	3.48 ± 0.469	3.09 ± 0.486	P = 0.057002
	5	Cosine	0.081 ± 0.022	0.064 ± 0.021	P = 0.052261
	5	Earth Mover's	14.64 ± 1.566	13.01 ± 1.920	P = 0.020457
	15	Jensen-Shannon Divergence	0.206 ± 0.024	0.223 ± 0.028	P = 0.101115
	15	Euclidean	4.28 ± 0.532	4.34 ± 0.763	P = 0.777432
	15	Cosine	0.091 ± 0.016	0.099 ± 0.028	P = 0.509539
	15	Earth Mover's	18.54 ± 2.717	18.68 ± 3.869	P = 1.00
	30	Jensen-Shannon Divergence	0.200 ± 0.028	0.234 ± 0.026	P = 0.002733
	30	Euclidean	3.78 ± 0.506	4.67 ± 0.907	P = 0.004988
	30	Cosine	0.085 ± 0.024	0.120 ± 0.035	P = 0.003490
	30	Earth Mover's	16.49 ± 2.87	20.68 ± 4.971	P = 0.010959
**AxB^{-3}**			**A^{-3} – AxB^{-3}**	**B^{-3} – AxB^{-3}**	
	1	Jensen-Shannon Divergence	0.263 ± 0.019	0.122 ± 0.013	P = 1.55 e-06
	1	Euclidean	4.45 ± 0.312	3.88 ± 0.402	P = 0.000490
	1	Cosine	0.116 ± 0.015	0.037 ± 0.007	P = 1.55 e-06
	1	Earth Mover's	19.65 ± 1.735	17.23 ± 2.26	P = 0.004434
	5	Jensen-Shannon Divergence	0.238 ± 0.012	0.125 ± 0.028	P = 1.55 e-06
	5	Euclidean	4.09 ± 0.679	4.00 ± 1.162	P = 0.664708
	5	Cosine	0.092 ± 0.013	0.039 ± 0.017	P = 2.25 e-06
	5	Earth Mover's	17.31 ± 2.728	17.97 ± 5.994	P = 0.865325
	15	Jensen-Shannon Divergence	0.300 ± 0.025	0.169 ± 0.038	P = 1.86 e-06

	15	Euclidean	4.05 ± 0.360	4.79 ± 0.79	*P* = 0.003490
	15	Cosine	0.152 ± 0.030	0.059 ± 0.022	*P* = 1.86 e-06
	15	Earth Mover's	16.79 ± 1.701	22.04 ± 4.227	*P* = 0.000319
	30	Jensen-Shannon Divergence	0.220 ± 0.022	0.200 ± 0.032	*P* = 0.101116
	30	Euclidean	4.33 ± 0.616	3.99 ± 0.794	*P* = 0.180911
	30	Cosine	0.090 ± 0.023	0.072 ± 0.022	*P* = 0.030226
	30	Earth Mover's	18.59 ± 2.389	17.26 ± 3.865	*P* = 0.235148
**AxB^{-5}**			**A^{-5} – AxB^{-5}**	**B^{-5} – AxB^{-5}**	
	1	Jensen-Shannon Divergence	0.116 ± 0.020	0.158 ± 0.020	*P* = 0.000059
	1	Euclidean	2.59 ± 0.323	2.97 ± 0.352	*P* = 0.005603
	1	Cosine	0.032 ± 0.007	0.042 ± 0.010	*P* = 0.003937
	1	Earth Mover's	10.53 ± 1.555	12.98 ± 1.411	*P* = 0.000177
	5	Jensen-Shannon Divergence	0.149 ± 0.024	0.163 ± 0.020	*P* = 0.146773
	5	Euclidean	2.88 ± 0.373	2.86 ± 0.231	*P* = 0.664708
	5	Cosine	0.046 ± 0.012	0.045 ± 0.007	*P* = 0.664708
	5	Earth Mover's	9.90 ± 1.374	12.31 ± 1.006	*P* = 0.000031
	15	Jensen-Shannon Divergence	0.145 ± 0.027	0.167 ± 0.025	*P* = 0.022597
	15	Euclidean	2.53 ± 0.420	2.74 ± 0.329	*P* = 0.117796
	15	Cosine	0.041 ± 0.011	0.045 ± 0.011	*P* = 0.266214
	15	Earth Mover's	9.45 ± 2.160	11.55 ± 0.935	*P* = 0.009832
	30	Jensen-Shannon Divergence	0.184 ± 0.019	0.177 ± 0.040	*P* = 0.9.54917
	30	Euclidean	2.95 ± 0.308	3.52 ± 0.774	*P* = 0.0522608
	30	Cosine	0.060 ± 0.013	0.047 ± 0.022	*P* = 0.0249297
	30	Earth Mover's	11.65 ± 1.653	14.95 ± 3.244	*P* = 0.0024137
**AxC^{-1}**			**A^{-1} – AxC^{-1}**	**C^{-1} – AxC^{-1}**	
	1	Jensen-Shannon Divergence	0.185 ± 0.031	0.110 ± 0.012	*P* = 1.86 e-06
	1	Euclidean	4.34 ± 0.575	2.62 ± 0.354	*P* = 1.86 e-06
	1	Cosine	0.068 ± 0.019	0.024 ± 0.007	*P* = 1.86 e-06
	1	Earth Mover's	17.36 ± 2.484	10.55 ± 1.034	*P* = 1.55 e-06
	5	Jensen-Shannon Divergence	0.234 ± 0.029	0.103 ± 0.020	*P* = 1.55 e-06
	5	Euclidean	5.57 ± 0.532	2.71 ± 0.531	*P* = 1.55 e-06
	5	Cosine	0.108 ± 0.020	0.022 ± 0.009	*P* = 1.55 e-06
	5	Earth Mover's	23.63 ± 2.887	11.46 ± 2.035	*P* = 1.55 e-06
	15	Jensen-Shannon Divergence	0.224 ± 0.019	0.100 ± 0.008	*P* = 1.55 e-06
	15	Euclidean	4.84 ± 0.331	2.36 ± 0.290	*P* = 1.55 e-06
	15	Cosine	0.092 ± 0.017	0.018 ± 0.005	*P* = 1.55 e-06
	15	Earth Mover's	21.47 ± 1.713	10.84 ± 1.412	*P* = 1.55 e-06
	30	Jensen-Shannon Divergence	0.236 ± 0.032	0.084 ± 0.011	*P* = 1.55 e-06
	30	Euclidean	5.14 ± 0.690	1.63 ± 0.182	*P* = 1.55 e-06
	30	Cosine	0.103 ± 0.026	0.011 ± 0.003	*P* = 1.55 e-06
	30	Earth Mover's	22.85 ± 4.043	7.07 ± 0.943	*P* = 1.55 e-06
**AxC^{-3}**			**A^{-3} – AxC^{-3}**	**C^{-3} – AxC^{-3}**	
	1	Jensen-Shannon Divergence	0.276 ± 0.018	0.081 ± 0.010	*P* = 1.55 e-06

	1	Euclidean	6.51 ± 0.406	2.02 ± 0.186	*P* = 1.55 e-06
	1	Cosine	0.139 ± 0.022	0.014 ± 0.003	*P* = 1.55 e-06
	1	Earth Mover's	30.15 ± 2.139	9.096 ± 1.065	*P* = 1.55 e-06
	5	Jensen-Shannon Divergence	0.274 ± 0.011	0.111 ± 0.009	*P* = 1.55 e-06
	5	Euclidean	4.88 ± 0.326	2.27 ± 0.240	*P* = 1.55 e-06
	5	Cosine	0.130 ± 0.008	0.022 ± 0.003	*P* = 1.55 e-06
	5	Earth Mover's	21.36 ± 1.831	10.13 ± 1.206	*P* = 1.55 e-06
	15	Jensen-Shannon Divergence	0.324 ± 0.014	0.088 ± 0.017	*P* = 1.55 e-06
	15	Euclidean	5.87 ± 0.471	2.18 ± 0.376	*P* = 1.55 e-06
	15	Cosine	0.171 ± 0.015	0.019 ± 0.005	*P* = 1.55 e-06
	15	Earth Mover's	26.13 ± 2.431	8.80 ± 1.521	*P* = 1.55 e-06
	30	Jensen-Shannon Divergence	0.290 ± 0.024	0.120 ± 0.015	*P* = 1.55 e-06
	30	Euclidean	6.98 ± 0.639	3.970 ± 0.555	*P* = 1.55 e-06
	30	Cosine	0.151 ± 0.028	0.037 ± 0.008	*P* = 1.55 e-06
	30	Earth Mover's	30.96 ± 3.762	17.95 ± 2.791	*P* = 1.55 e-06
**AxC^{-5}**			**A^{-5} – AxC^{-5}**	**C^{-5} – AxC^{-5}**	
	1	Jensen-Shannon Divergence	0.150 ± 0.033	0.199 ± 0.019	*P* = 0.000238
	1	Euclidean	2.97 ± 0.650	4.42 ± 0.305	*P* = 3.90 e-06
	1	Cosine	0.043 ± 0.018	0.088 ± 0.015	*P* = 9.49 e-06
	1	Earth Mover's	11.72 ± 2.587	20.45 ± 2.340	*P* = 1.55 e-06
	5	Jensen-Shannon Divergence	0.199 ± 0.013	0.204 ± 0.009	*P* = 0.336518
	5	Euclidean	4.17 ± 0.4536435	6.07 ± 0.331	*P* = 1.55 e-06
	5	Cosine	0.074 ± 0.012	0.094 ± 0.009	*P* = 0.000060
	5	Earth Mover's	16.24 ± 2.189	31.08 ± 2.289	*P* = 1.55 e-06
	15	Jensen-Shannon Divergence	0.199 ± 0.021	0.187 ± 0.016	*P* = 0.126910
	15	Euclidean	3.68 ± 0.458	3.78 ± 0.373	*P* = 0.584730
	15	Cosine	0.067 ± 0.017	0.072 ± 0.013	*P* = 0.417761
	15	Earth Mover's	14.60 ± 1.548	16.04 ± 2.087	*P* = 0.101116
	30	Jensen-Shannon Divergence	0.225 ± 0.016	0.197 ± 0.017	*P* = 0.000152
	30	Euclidean	3.76 ± 0.295	3.77 ± 0.461	*P* = 0.835784
	30	Cosine	0.085 ± 0.012	0.084 ± 0.018	*P* = 0.924933
	30	Earth Mover's	14.06956 1.249893	16.74787 1.822435	*P* = 0.000205
**BxC^{-1}**			**B^{-1} – BxC^{-1}**	**C^{-1} – BxC^{-1}**	
	1	Jensen-Shannon Divergence	0.222 ± 0.011	0.113 ± 0.010	*P* = 1.55 e-06
	1	Euclidean	5.68 ± 0.301	2.59 ± 0.105	*P* = 1.55 e-06
	1	Cosine	0.110 ± 0.012	0.024 ± 0.003	*P* = 1.55 e-06
	1	Earth Mover's	26.57 ± 1.742	10.62 ± 0.505	*P* = 1.55 e-06
	5	Jensen-Shannon Divergence	0.221 ± 0.012	0.138 ± 0.013	*P* = 1.55 e-06
	5	Euclidean	5.762 ± 0.537	3.287 ± 0.383	*P* = 1.55 e-06
	5	Cosine	0.110 ± 0.020	0.030 ± 0.005	*P* = 1.55 e-06
	5	Earth Mover's	25.73 ± 2.757	14.79 ± 1.851	*P* = 1.55 e-06
	15	Jensen-Shannon Divergence	0.248 ± 0.026	0.140 ± 0.013	*P* = 1.55 e-06
	15	Euclidean	5.92 ± 0.720	3.55 ± 0.290	*P* = 1.55 e-06

	15	Cosine	0.123 ± 0.036	0.036 ± 0.006	*P* = 1.55 e-06
	15	Earth Mover's	28.26 ± 3.875	16.58 ± 1.415	*P* = 1.55 e-06
	30	Jensen-Shannon Divergence	0.260 ± 0.021	0.091 ± 0.016	*P* = 1.55 e-06
	30	Euclidean	6.74 ± 0.965	2.19 ± 0.434	*P* = 1.55 e-06
	30	Cosine	0.144 ± 0.027	0.018 ± 0.006	*P* = 1.55 e-06
	30	Earth Mover's	32.79 ± 5.501	9.28 ± 1.560	*P* = 1.55 e-06
**BxC^{-3}**			**B^{-3} – BxC^{-3}**	**C^{-3} – BxC^{-3}**	
	1	Jensen-Shannon Divergence	0.172 ± 0.012	0.129 ± 0.014	*P* = 1.55 e-06
	1	Euclidean	3.97 ± 0.140	2.76 ± 0.234	*P* = 1.55 e-06
	1	Cosine	0.055 ± 0.007	0.031 ± 0.005	*P* = 1.55 e-06
	1	Earth Mover's	18.32 ± 0.649	12.42 ± 1.186	*P* = 1.55 e-06
	5	Jensen-Shannon Divergence	0.132 ± 0.010	0.149 ± 0.019	*P* = 0.004988
	5	Euclidean	3.38 ± 0.122	3.25 ± 0.512	*P* = 0.235148
	5	Cosine	0.041 ± 0.003	0.039 ± 0.012	*P* = 0.250345
	5	Earth Mover's	14.13 ± 1.208	13.47 ± 2.322	*P* = 0.282762
	15	Jensen-Shannon Divergence	0.190 ± 0.016	0.095 ± 0.016	*P* = 1.55 e-06
	15	Euclidean	4.27 ± 0.295	2.44 ± 0.413	*P* = 1.55 e-06
	15	Cosine	0.077 ± 0.013	0.025 ± 0.008	*P* = 1.55 e-06
	15	Earth Mover's	18.29 ± 1.218	10.03 ± 1.763	*P* = 1.55 e-06
	30	Jensen-Shannon Divergence	0.245 ± 0.017	0.117 ± 0.015	*P* = 1.55 e-06
	30	Euclidean	5.91 ± 0.809	3.36 ± 0.618	*P* = 1.55 e-06
	30	Cosine	0.120 ± 0.023	0.032 ± 0.006	*P* = 1.55 e-06
	30	Earth Mover's	27.32 ± 4.970	14.91 ± 2.727	*P* = 1.55 e-06
**BxC^{-5}**			**B^{-5} – BxC^{-5}**	**C^{-5} – BxC^{-5}**	
	1	Jensen-Shannon Divergence	0.239 ± 0.026	0.176 ± 0.009	*P* = 1.55 e-06
	1	Euclidean	5.46 ± 0.791	5.82 ± 0.557	*P* = 0.157557
	1	Cosine	0.103 ± 0.031	0.071 ± 0.011	*P* = 0.003091
	1	Earth Mover's	22.68 ± 3.300	28.15 ± 3.563	*P* = 0.000852
	5	Jensen-Shannon Divergence	0.234 ± 0.020	0.215 ± 0.010	*P* = 0.004434
	5	Euclidean	5.52 ± 0.450	7.49 ± 0.293	*P* = 1.55 e-06
	5	Cosine	0.102 ± 0.019	0.103 ± 0.013	*P* = 0.748699
	5	Earth Mover's	23.82 ± 2.478	38.01 ± 1.4921	*P* = 1.55 e-06
	15	Jensen-Shannon Divergence	0.273 ± 0.015	0.202 ± 0.013	*P* = 1.55 e-06
	15	Euclidean	5.88 ± 0.514	5.02 ± 0.496	*P* = 0.000205
	15	Cosine	0.130 ± 0.017	0.078 ± 0.011	*P* = 1.55 e-06
	15	Earth Mover's	24.70 ± 1.916	22.82 ± 3.025	*P* = 0.086374
	30	Jensen-Shannon Divergence	0.258 ± 0.033	0.189 ± 0.025	*P* = 0.000013
	30	Euclidean	5.00 ± 0.968	4.56 ± 0.636	*P* = 0.180911
	30	Cosine	0.108 ± 0.034	0.080 ± 0.018	*P* = 0.012200
	30	Earth Mover's	21.04 ± 3.741	20.40 ± 3.441	*P* = 0.534029

Quantification of donor contributions to outcome enzymatic activities following MCC

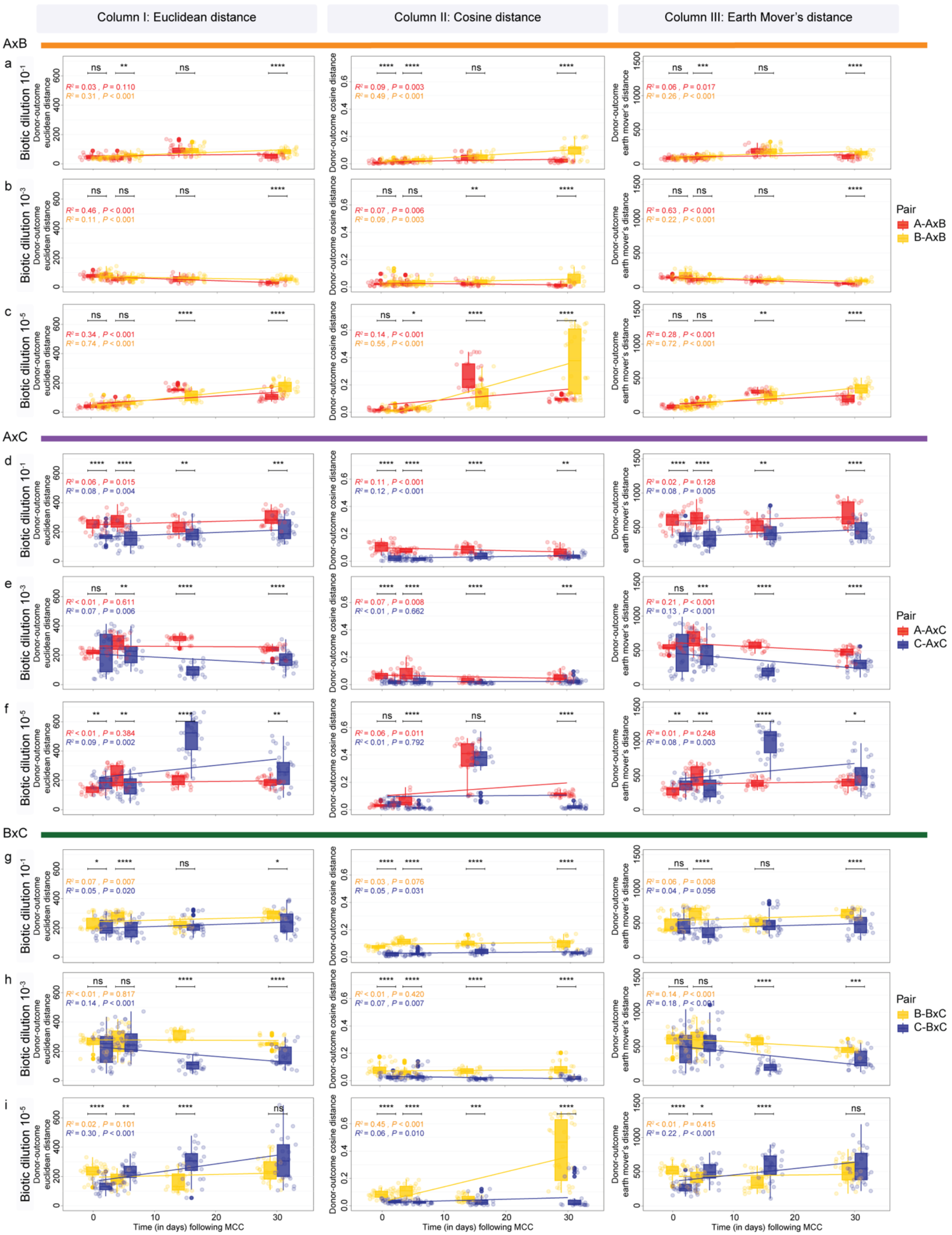

Supplementary Figure S8. Boxplots and linear regressions displaying the relative contribution of donors to the outcome enzymatic activities based on Euclidean (Column I), Cosine (Column II), and Earth Mover's distances (Column III) over time based on donor-outcome distances (**a**) AxB at the biotic dilution 10^{-1}, (**b**) AxB at the biotic dilution 10^{-3}, (**c**) AxB at the biotic dilution 10^{-5}, (**d**) AxC at the biotic dilution 10^{-1}, (**e**) AxC at the biotic dilution 10^{-3}, (**f**) AxC at the biotic dilution 10^{-5}, (**g**) BxC at the biotic dilution 10^{-1}, (**h**) BxC at the biotic dilution 10^{-3}, and (**i**) BxC at the biotic dilution 10^{-5}. Statistical differences in the relative contributions of donors to outcome were assessed by non-parametric Mann Whitney U and are denoted by an asterisk above the boxplot ("ns", $P > 0.05$; "*", $P < 0.05$; "**", $P < 0.01$; "***", $P < 0.001$; "****", $P < 0.0001$). Lower distances represent a greater contribution of the donor to the outcome community (i.e., dominance).

Supplementary Table S8. Contribution of donors to outcome enzymatic activity based on Jensen-Shannon divergence, Euclidean, Cosine, and Earth Mover's distances over time. Values are means ± SD; n = 288. Statistical differences were determined using non-parametric Mann Whitney U test at $\alpha = 0.05$.

**Outcome Enzymatic activity**	**Time (days)**	**Distances**	**Donor – Outcome**		**Mann Whitney U test**
**AxB^{-1}**			**A^{-1} – AxB^{-1}**	**B^{-1} – AxB^{-1}**	
	1	Jensen-Shannon Divergence	0.155 ± 0.043	0.198 ± 0.053	P = 0.004084
	1	Euclidean	48.04 ± 16.770	38.24 ± 7.395	P = 0.054747
	1	Cosine	0.010 ± 0.008	0.022 ± 0.010	P = 0.000015
	1	Earth Mover's	85.66 ± 26.348	88.28 ± 18.456	P = 0.613928
	5	Jensen-Shannon Divergence	0.166 ± 0.044	0.202 ± 0.058	P = 0.010962
	5	Euclidean	42.55 ± 18.948	56.78 ± 16.673	P = 0.004636
	5	Cosine	0.013 ± 0.009	0.028 ± 0.014	P = 0.000033
	5	Earth Mover's	83.05 ± 28.090	110.23 ± 27.70	P = 0.000620
	15	Jensen-Shannon Divergence	0.281 ± 0.048	0.250 ± 0.050	P = 0.035341
	15	Euclidean	92.88 ± 33.002	93.29 ± 27.265	P = 0.939369
	15	Cosine	0.049 ± 0.029	0.052 ± 0.024	P = 0.462167
	15	Earth Mover's	185.26 ± 58.182	195.86 ± 55.883	P = 0.483005
	30	Jensen-Shannon Divergence	0.223 ± 0.069	0.301 ± 0.065	P = 0.000036
	30	Euclidean	52.06 ± 16.324	82.04 ± 22.109	P = 9.70 e-06
	30	Cosine	0.023 ± 0.012	0.104 ± 0.059	P = 2.42 e-06
	30	Earth Mover's	106.23 ± 32.835	159.13 ± 38.913	P = 0.000022
**AxB^{-3}**			**A^{-3} – AxB^{-3}**	**B^{-3} – AxB^{-3}**	
	1	Jensen-Shannon Divergence	0.155 ± 0.046	0.173 ± 0.069	P = 0.351679
	1	Euclidean	78.04 ± 16.823	79.88 ± 28.50	P = 0.669479
	1	Cosine	0.025 ± 0.019	0.037 ± 0.034	P = 0.393257
	1	Earth Mover's	144.54 ± 18.215	170.62 ± 61.78	P = 0.268743
	5	Jensen-Shannon Divergence	0.119 ± 0.039	0.096 ± 0.027	P = 0.039715
	5	Euclidean	55.67 ± 17.380	58.71 ± 17.705	P = 0.497075
	5	Cosine	0.030 ± 0.024	0.034 ± 0.024	P = 0.509448
	5	Earth Mover's	112.45 ± 26.88	113.10 ± 35.776	P = 0.907322
	15	Jensen-Shannon Divergence	0.212 ± 0.044	0.193 ± 0.034	P = 0.130172

	15	Euclidean	52.56 ± 20.410	59.29 ± 17.081	*P* = 0.187038
	15	Cosine	0.026 ± 0.016	0.040 ± 0.017	*P* = 0.002991
	15	Earth Mover's	99.89 ± 30.739	106.73 ± 22.593	*P* = 0.331976
	30	Jensen-Shannon Divergence	0.121 ± 0.052	0.172 ± 0.052	*P* = 0.001785
	30	Euclidean	29.66 ± 10.966	54.30 ± 15.048	*P* = 7.51 e-07
	30	Cosine	0.014 ± 0.012	0.060 ± 0.042	*P* = 1.50 e-06
	30	Earth Mover's	52.11 ± 18.013	93.78 ± 24.544	*P* = 2.21 e-07
**AxB^{-5}**			**A^{-5} – AxB^{-5}**	**B^{-5} – AxB^{-5}**	
	1	Jensen-Shannon Divergence	0.155 ± 0.049	0.129 ± 0.061	*P* = 0.057239
	1	Euclidean	41.31 ± 12.741	46.06 ± 25.402	*P* = 0.846154
	1	Cosine	0.018 ± 0.008	0.019 ± 0.019	*P* = 0.125319
	1	Earth Mover's	83.69 ± 24.373	80.101 ± 39.756	*P* = 0.437682
	5	Jensen-Shannon Divergence	0.131 ± 0.042	0.184 ± 0.042	*P* = 0.000089
	5	Euclidean	56.186 ± 18.500	61.05 ± 17.195	*P* = 0.214317
	5	Cosine	0.021 ± 0.011	0.028 ± 0.011	*P* = 0.025659
	5	Earth Mover's	126.842 ± 37.549	138.78 ± 36.902	*P* = 0.130172
	15	Jensen-Shannon Divergence	0.377 ± 0.042	0.188 ± 0.059	*P* = 2.29 e-09
	15	Euclidean	160.00 ± 17.205	111.67 ± 35.900	*P* = 4.26 e-06
	15	Cosine	0.271 ± 0.108	0.113 ± 0.081	*P* = 2.92 e-06
	15	Earth Mover's	300.42 ± 33.134	236.67 ± 75.625	*P* = 0.001906
	30	Jensen-Shannon Divergence	0.293 ± 0.015	0.417 ± 0.106	*P* = 0.000098
	30	Euclidean	108.41 ± 26.767	174.86 ± 38.398	*P* = 1.06 e-07
	30	Cosine	0.098 ± 0.018	0.376 ± 0.231	*P* = 2.96 e-06
	30	Earth Mover's	201.24 ± 64.506	348.51 ± 84.686	*P* = 2.02 e-07
**AxC^{-1}**			**A^{-1} – AxC^{-1}**	**C^{-1} – AxC^{-1}**	
	1	Jensen-Shannon Divergence	0.303 ± 0.043	0.111 ± 0.050	*P* = 1.42 e-09
	1	Euclidean	249.25 ± 38.421	175.16 ± 44.073	*P* = 1.65 e-06
	1	Cosine	0.108 ± 0.036	0.025 ± 0.020	*P* = 5.21 e-09
	1	Earth Mover's	599.30 ± 111.38	378.75 ± 104.401	*P* = 4.10 e-07
	5	Jensen-Shannon Divergence	0.269 ± 0.037	0.098 ± 0.039	*P* = 1.42 e-09
	5	Euclidean	276.11 ± 52.666	162.43 ± 65.232	*P* = 4.54 e-07
	5	Cosine	0.076 ± 0.015	0.016 ± 0.012	*P* = 1.42 e-09
	5	Earth Mover's	646.98 ± 129.546	337.34 ± 134.52	*P* = 2.87 e-08
	15	Jensen-Shannon Divergence	0.303 ± 0.034	0.166 ± 0.051	*P* = 1.80 e-09
	15	Euclidean	233.70 ± 45.382	186.22 ± 60.382	*P* = 0.002471
	15	Cosine	0.091 ± 0.030	0.040 ± 0.023	*P* = 4.67 e-06
	15	Earth Mover's	533.94 ± 105.201	428.14 ± 160.880	*P* = 0.002635
	30	Jensen-Shannon Divergence	0.260 ± 0.056	0.178 ± 0.039	*P* = 2.66 e-06
	30	Euclidean	296.39 ± 52.494	214.81 ± 73.436	*P* = 0.000227
	30	Cosine	0.066 ± 0.036	0.038 ± 0.017	*P* = 0.003609
	30	Earth Mover's	685.94 ± 176.354	454.28 ± 136.640	*P* = 0.000070
**AxC^{-3}**			**A^{-3} – AxC^{-3}**	**C^{-3} – AxC^{-3}**	
	1	Jensen-Shannon Divergence	0.158 ± 0.023	0.081 ± 0.035	*P* = 2.83 e-09

	1	Euclidean	217.61 ± 24.349	222.87 ± 131.763	*P* = 0.939368
	1	Cosine	0.064 ± 0.021	0.021 ± 0.017	*P* = 9.55 e-09
	1	Earth Mover's	541.25 ± 59.408	500.35 ± 309.657	*P* = 0.952824
	5	Jensen-Shannon Divergence	0.170 ± 0.043	0.083 ± 0.033	*P* = 4.00 e-08
	5	Euclidean	284.91 ± 56.282	222.52 ± 85.635	*P* = 0.007415
	5	Cosine	0.084 ± 0.050	0.027 ± 0.022	*P* = 4.26 e-06
	5	Earth Mover's	666.00 ± 140.898	466.61 ± 200.900	*P* = 0.000594
	15	Jensen-Shannon Divergence	0.240 ± 0.045	0.127 ± 0.018	*P* = 1.80 e-09
	15	Euclidean	308.22 ± 28.434	97.99 ± 41.007	*P* = 1.42 e-09
	15	Cosine	0.031 ± 0.016	0.011 ± 0.007	*P* = 1.12 e-06
	15	Earth Mover's	566.93 ± 59.539	187.05 ± 66.749	*P* = 1.42 e-09
	30	Jensen-Shannon Divergence	0.181 ± 0.060	0.130 ± 0.048	*P* = 0.000856
	30	Euclidean	233.91 ± 34.212	171.00 ± 57.881	*P* = 0.000013
	30	Cosine	0.052 ± 0.028	0.026 ± 0.021	*P* = 0.000149
	30	Earth Mover's	464.68 ± 84.043	308.39 ± 99.367	*P* = 6.53 e-07
**AxC^{-5}**			**A^{-5} – AxC^{-5}**	**C^{-5} – AxC^{-5}**	
	1	Jensen-Shannon Divergence	0.194 ± 0.043	0.169 ± 0.039	*P* = 0.057239
	1	Euclidean	138.76 ± 27.239	176.84 ± 51.954	*P* = 0.005206
	1	Cosine	0.034 ± 0.017	0.045 ± 0.028	*P* = 0.244355
	1	Earth Mover's	285.76 ± 71.095	361.71 ± 101.267	*P* = 0.006997
	5	Jensen-Shannon Divergence	0.200 ± 0.048	0.144 ± 0.034	*P* = 0.000039
	5	Euclidean	232.13 ± 70.639	156.342 ± 71.417	*P* = 0.001194
	5	Cosine	0.065 ± 0.038	0.016 ± 0.015	*P* = 8.55 e-08
	5	Earth Mover's	495.51 ± 148.314	328.35 ± 148.726	*P* = 0.000553
	15	Jensen-Shannon Divergence	0.429 ± 0.061	0.376 ± 0.090	*P* = 0.012316
	15	Euclidean	204.06 ± 38.461	500.78 ± 124.673	*P* = 8.28 e-09
	15	Cosine	0.368 ± 0.147	0.337 ± 0.146	*P* = 0.221564
	15	Earth Mover's	385.78 ± 64.494	998.32 ± 217.142	*P* = 2.57 e-09
	30	Jensen-Shannon Divergence	0.307 ± 0.014	0.169 ± 0.026	*P* = 3.02 e-11
	30	Euclidean	186.20 ± 31.216	266.17 ± 117.143	*P* = 0.003848
	30	Cosine	0.113 ± 0.017	0.021 ± 0.015	*P* = 3.02 e-11
	30	Earth Mover's	402.47 ± 69.980	519.48 ± 213.854	*P* = 0.028129
**BxC^{-1}**			**B^{-1} – BxC^{-1}**	**C^{-1} – BxC^{-1}**	
	1	Jensen-Shannon Divergence	0.199 ± 0.020	0.118 ± 0.034	*P* = 3.67 e-09
	1	Euclidean	232.31 ± 49.415	207.31 ± 62.866	*P* = 0.026972
	1	Cosine	0.073 ± 0.016	0.022 ± 0.014	*P* = 1.60 e-09
	1	Earth Mover's	484.84 ± 123.268	452.81 ± 147.612	*P* = 0.116036
	5	Jensen-Shannon Divergence	0.249 ± 0.036	0.112 ± 0.044	*P* = 8.03 e-10
	5	Euclidean	279.35 ± 29.083	190.79 ± 54.696	*P* = 1.53 e-07
	5	Cosine	0.115 ± 0.020	0.028 ± 0.019	*P* = 2.45 e-10
	5	Earth Mover's	650.93 ± 81.581	363.33 ± 98.815	*P* = 6.49 e-10
	15	Jensen-Shannon Divergence	0.294 ± 0.031	0.179 ± 0.050	*P* = 1.68 e-09
	15	Euclidean	220.39 ± 33.074	216.16 ± 53.899	*P* = 0.441840

	15	Cosine	0.105 ± 0.027	0.046 ± 0.023	*P* = 8.64 e-09
	15	Earth Mover's	496.17 ± 69.825	499.35 ± 151.301	*P* = 0.233392
	30	Jensen-Shannon Divergence	0.266 ± 0.022	0.146 ± 0.023	*P* = 1.42 e-09
	30	Euclidean	290.59 ± 27.732	238.60 ± 84.371	*P* = 0.011030
	30	Cosine	0.103 ± 0.036	0.033 ± 0.013	*P* = 4.13 e-09
	30	Earth Mover's	648.16 ± 66.048	477.90 ± 142.873	*P* = 0.000028
**BxC^{-3}**			**B^{-3} – BxC^{-3}**	**C^{-3} – BxC^{-3}**	
	1	Jensen-Shannon Divergence	0.166 ± 0.032	0.096 ± 0.033	*P* = 2.10 e-08
	1	Euclidean	257.72 ± 49.070	221.17 ± 114.981	*P* = 0.365828
	1	Cosine	0.083 ± 0.041	0.028 ± 0.018	*P* = 3.76 e-08
	1	Earth Mover's	583.01 ± 132.665	485.82 ± 266.729	*P* = 0.114007
	5	Jensen-Shannon Divergence	0.168 ± 0.038	0.110 ± 0.038	*P* = 8.84 e-07
	5	Euclidean	276.45 ± 88.403	261.50 ± 89.188	*P* = 0.395267
	5	Cosine	0.063 ± 0.032	0.035 ± 0.032	*P* = 0.000023
	5	Earth Mover's	608.55 ± 188.758	574.75 ± 220.807	*P* = 0.347828
	15	Jensen-Shannon Divergence	0.233 ± 0.034	0.114 ± 0.037	*P* = 6.57 e-09
	15	Euclidean	316.31 ± 33.013	105.56 ± 37.360	*P* = 1.42 e-09
	15	Cosine	0.069 ± 0.022	0.013 ± 0.008	*P* = 1.60 e-09
	15	Earth Mover's	572.04 ± 78.802	200.07 ± 71.773	*P* = 1.42 e-09
	30	Jensen-Shannon Divergence	0.233 ± 0.080	0.155 ± 0.059	*P* = 0.000973
	30	Euclidean	254.72 ± 35.029	159.18 ± 66.084	*P* = 9.70 e-06
	30	Cosine	0.083 ± 0.053	0.019 ± 0.014	*P* = 5.03 e-07
	30	Earth Mover's	455.80 ± 76.446	306.17 ± 128.558	*P* = 0.000132
**BxC^{-5}**			**B^{-5} – BxC^{-5}**	**C^{-5} – BxC^{-5}**	
	1	Jensen-Shannon Divergence	0.251 ± 0.037	0.187 ± 0.028	*P* = 3.65 e-08
	1	Euclidean	231.80 ± 50.325	132.94 ± 39.300	*P* = 2.83 e-08
	1	Cosine	0.083 ± 0.028	0.032 ± 0.013	*P* = 1.29 e-09
	1	Earth Mover's	505.61 ± 102.315	272.01 ± 85.272	*P* = 2.44 e-09
	5	Jensen-Shannon Divergence	0.269 ± 0.057	0.192 ± 0.028	*P* = 4.67 e-06
	5	Euclidean	176.04 ± 47.321	227.76 ± 61.251	*P* = 0.003609
	5	Cosine	0.107 ± 0.047	0.026 ± 0.013	*P* = 5.86 e-09
	5	Earth Mover's	397.06 ± 112.530	499.11 ± 142.657	*P* = 0.015293
	15	Jensen-Shannon Divergence	0.195 ± 0.038	0.149 ± 0.050	*P* = 0.002808
	15	Euclidean	163.37 ± 66.468	292.74 ± 106.565	*P* = 0.000026
	15	Cosine	0.054 ± 0.025	0.033 ± 0.032	*P* = 0.000907
	15	Earth Mover's	343.32 ± 124.426	569.44 ± 205.984	*P* = 0.000104
	30	Jensen-Shannon Divergence	0.448 ± 0.104	0.183 ± 0.069	*P* = 1.23 e-09
	30	Euclidean	244.17 ± 78.295	322.19 ± 153.232	*P* = 0.071832
	30	Cosine	0.411 ± 0.220	0.064 ± 0.098	*P* = 1.91 e-08
	30	Earth Mover's	507.94 ± 175.024	591.00 ± 262.038	*P* = 0.348182

Quantifying the importance of abiotic variables modulating biotic community coalescence

Supplementary Table S9. Results of the Random Forest regression analysis showing the importance of abiotic variables in explaining the variation in community structure following MCC. Variable importance was estimated by calculating the mean decrease in accuracy (%INCMSE) determined by permuting the values of each variable of the dataset and comparing these to the unpermuted set. Higher %IncMSE values represent higher variable importance. Mean decrease Gini (IncNodePurity) was assessed to measure the quality of each variable in the model. Higher IncNodePurity value represents a higher variable importance and is often aligned with permutated values. Estimated *P*-values were calculated by permutation (%IncMSE.pval) and IncNodePurity (IncNodePurity.pvals) at α = 0.05, and statistically significant %IncMSE and IncNodePurity values are presented in bold. The total explained variance of the random forest regression model for each treatment is represented between parenthesis following the biotic dilution treatment.

	**Treatment**	**%IncMSE**	**%IncMSE.pval**	**IncNodePurity**	**IncNodePurity.pvals**
	**Soils AxB**				
**Predictor**	**Biotic dilution 10^{-1} (43.21%)**				
Sand		9.30291	0.10891089	0.1003069	0.78217822
pH		**12.24033**	0.04950495	**0.2035866**	0.00990099
Organic matter		11.34061	0.06930693	0.1395284	0.14851485
NH_4^+		**18.13138**	0.00990099	**0.6679871**	0.00990099
NO_3^-		**22.53745**	0.01980198	**0.8691988**	0.00990099
Total N		**16.76303**	0.01980198	**0.5559387**	0.00990099
Sulfur		**14.83332**	0.00990099	**0.4826764**	0.00990099
	**Biotic dilution 10^{-3} (22.86%)**				
Sand		1.806375	0.40594059	0.01515611	0.97029703
pH		6.716813	0.22772277	**0.03025580**	0.02970297
Organic matter		8.065067	0.14851485	0.02002657	0.45544554
NH_4^+		**15.765750**	0.00990099	**0.07870826**	0.00990099
NO_3^-		**19.867744**	0.01980198	**0.10234110**	0.00990099
Total N		**15.652642**	0.00990099	**0.06414147**	0.00990099
Sulfur		**16.236370**	0.00990099	**0.07374127**	0.00990099
	**Biotic dilution 10^{-5} (36.71%)**				
Sand		-11.823330	0.96039604	0.01264035	1.00000000
pH		6.638418	0.19801980	**0.05980536**	0.00990099
Organic matter		-3.484455	0.63366337	0.02136281	0.97029703
NH_4^+		**16.824279**	0.01980198	**0.17741804**	0.00990099
NO_3^-		**16.782255**	0.00990099	**0.17044842**	0.00990099
Total N		**17.315898**	0.00990099	**0.16921174**	0.00990099
Sulfur		**15.943227**	0.00990099	**0.15069320**	0.00990099
	**Soils AxC**				
Predictor	**Biotic dilution 10^{-1} (96.86%)**				
Sand		7.158460	0.22772277	0.2862118	0.93069307
pH		**24.573036**	0.00990099	**6.5575010**	0.00990099
Organic matter		9.380794	0.16831683	0.5597604	0.06930693
NH_4^+		**26.831334**	0.00990099	**7.4491797**	0.00990099
NO_3^-		**16.360496**	0.01980198	**2.6287404**	0.00990099
Total N		**16.898294**	0.00990099	**2.7623064**	0.00990099
Sulfur		11.400447	0.08910891	**0.7812611**	0.01980198
	**Biotic dilution 10^{-3}**				

	**(95.48%)**				
Sand		7.626390	0.14851485	0.2363507	0.76237624
pH		**26.776106**	0.00990099	**5.4257289**	0.00990099
Organic matter		9.619217	0.10891089	0.3718658	0.06930693
NH_4^+		**25.817154**	0.00990099	**4.9837639**	0.00990099
NO_3^-		**15.236950**	0.00990099	**1.5185773**	0.00990099
Total N		**15.384642**	0.00990099	**1.5747808**	0.00990099
Sulfur		10.411565	0.09900990	**0.5564700**	0.01980198
	**Biotic dilution 10^{-5} (91.31%)**				
Sand		7.876513	0.11881188	0.1881542	0.89108911
pH		**25.803696**	0.00990099	**3.5863843**	0.00990099
Organic matter		9.896413	0.11881188	0.2507245	0.34653465
NH_4^+		**27.057885**	0.00990099	**3.7863524**	0.00990099
NO_3^-		**15.303569**	0.00990099	**1.0932345**	0.00990099
Total N		**16.622638**	0.01980198	**1.4405361**	0.00990099
Sulfur		10.567491	0.10891089	**0.4083231**	0.04950495
	**Soils BxC**				
Predictor	**Biotic dilution 10^{-1} (97.81%)**				
Sand		3.471012	0.33663366	0.07349318	1.00000000
pH		**20.136408**	0.00990099	**6.81714975**	0.00990099
Organic matter		6.355201	0.20792079	0.36649793	0.96039604
NH_4^+		**20.342252**	0.00990099	**6.78630949**	0.00990099
NO_3^-		**12.836491**	0.04950495	**2.08892688**	0.00990099
Total N		**12.850484**	0.02970297	**2.24267539**	0.00990099
Sulfur		**22.481088**	0.00990099	**7.89111847**	0.00990099
	**Biotic dilution 10^{-3} (97.71%)**				
Sand		3.838213	0.29702970	0.07569075	1.00000000
pH		**22.871591**	0.00990099	**6.83705370**	0.00990099
Organic matter		6.717879	0.14851485	0.31230813	0.88118812
NH_4^+		**20.802781**	0.00990099	**5.83501872**	0.00990099
NO_3^-		**12.489405**	0.02970297	**1.55387427**	0.00990099
Total N		**13.205249**	0.00990099	**1.96105473**	0.00990099
Sulfur		**18.786250**	0.00990099	**5.20675888**	0.00990099
	**Biotic dilution 10^{-5} (79.38%)**				
Sand		0.9905272	0.42574257	0.04763705	1.00000000
pH		**21.7793058**	0.01980198	**2.62899166**	0.00990099
Organic matter		4.6804463	0.24752475	0.14197223	0.96039604
NH_4^+		**20.9381971**	0.00990099	**2.44558410**	0.00990099
NO_3^-		**12.8729314**	0.04950495	**0.74367129**	0.00990099
Total N		**12.0545364**	0.03960396	**0.64945899**	0.00990099
Sulfur		**18.7059958**	0.00990099	**2.12410783**	0.00990099

Supplementary References

1. Gee GW, Bauder JW. Particle size analysis. In: Amer. Soc. Agron. (ed.), Methods of Soil Analysis: Part 1. Physical and Mineralogical Methods, 2nd ed. Madison, WI, USA, 1986, 383–411.
2. Eckert D, Sims JT. Recommended Soil pH and Lime Requirement Tests. Recommended Soil Testing Procedures for the Northeastern United States. Newark, DE, 2011, 19–25.
3. Mehlich A. New buffer pH method for rapid estimation of exchangeable acidity and lime requirement of soils. *Commun Soil Sci Plant Anal* 1976;**7**:637–52. https://doi.org/10.1080/00103627609366673
4. Wolf A, Beegle D. Recommended soil tests for macro and micronutrients. *Recommended Soil Testing Procedures for the Northeastern United States* . 2011.
5. Ross D, Kettering Q. Recommended methods for determining soil cation exchange capacity. Chapter 9. *Recommended Soil Testing Procedures for the Northeasten United States Cooperative Bulletin No 493* 2011;75–86.
6. Schulte EE, Hoskins B. Recommended Soil Organic Matter Tests. *Recommended Soil Testing Procedures for the Northeastern United States* 2011;63–74.
7. Nelson DW, Sommers LE. Total Carbon, Organic Carbon, and Organic Matter. Methods of Soil Analysis, Part 3: Chemical Methods. Madison, WI, USA: wiley, 2018, 961–1010.
8. Gartley K. Recommended Soluble Salts Tests. Recommended Soil Testing Procedures for the Northeastern United States, Northeast. Newark, DE, 2011, 87–94.
9. Griffin G et al. Recommended Soil Nitrate-N Tests. *Recommended Soil Testing Procedures for the Northeastern United States* 2011;27–38.
10. Gavlak R et al. Soil Ammonium Nitrogen. Soil, Plant and Water Reference Methods for the Western Region, 3rd ed. Western Region Extension Publication, 2005, 65–66.
11. Bremner JM. Nitrogen-Total. Methods of Soil Analysis, Part 3: Chemical Methods. Madison, WI, USA, 2018, 1085–1121.
12. Pella E. Elemental organic-analysis. 1. Historical developments. *Am Lab* 1990;**22**:116.
13. German DP et al. Optimization of hydrolytic and oxidative enzyme methods for ecosystem studies. *Soil Biol Biochem* 2011;**43**:1387–97. https://doi.org/10.1016/j.soilbio.2011.03.017
14. DeForest JL. The influence of time, storage temperature, and substrate age on potential soil enzyme activity in acidic forest soils using MUB-linked substrates and l-DOPA. *Soil Biol Biochem* 2009;**41**:1180–6. https://doi.org/10.1016/j.soilbio.2009.02.029
